# Epidemiologic Investigation and Genetic Variation Analysis of PRRSV, PCV2, and PCV3 in Guangdong Province, China from 2020 to 2022

**DOI:** 10.3390/v16111687

**Published:** 2024-10-29

**Authors:** Zhongmao Yuan, Yawei Sun, Xinni Niu, Quanhui Yan, Weijun Zeng, Pengfei Du, Kaiyuan Xie, Yiqi Fang, Lianxiang Wang, Hongxing Ding, Lin Yi, Mingqiu Zhao, Shuangqi Fan, Dongfang Zhao, Jinding Chen

**Affiliations:** 1 College of Veterinary Medicine, South China Agricultural University, No. 483 Wushan Road, Tianhe District, Guangzhou 510642, China; 13073059918@163.com (Z.Y.); syw18530494979@163.com (Y.S.);; 2 Wen’s Group Academy, Wen’s Foodstuffs Group Co., Ltd., Yunfu 527400, China; 3 Yunfu Branch, Guangdong Laboratory for Lingnan Modern Agriculture, HuiNeng North Road, Yunfu 527400, China

**Keywords:** porcine reproductive and respiratory syndrome virus (PRRSV), porcine circovirus type 2 (PCV2), porcine circovirus type 3 (PCV3), epidemiological investigation, genetic variation analysis

## Abstract

Recently, the emergence of HP-PRRSV (Highly Pathogenic porcine reproductive and respiratory syndrome virus) and the exacerbation of mixed infections of PRRSV and PCV have resulted in significant economic losses for the Chinese pig industry. This study collected a total of 226 samples suspected of infection with the aforementioned viruses from diverse pig farms in seven urban districts of central and northern Guangdong Province between 2020 and 2022. The positive rates of PRRSV, PCV2, and PCV3 in the samples were 33.2%, 37.6%, and 7.5%, respectively, and there were various mixed-infection scenarios present in the samples. This study successfully isolated multiple strains of PRRSV2 and PCV2 from their positive samples, and obtained the gene sequences of six PCV3 (*ORF1* + *ORF2*) from samples. The associated sequences obtained were subjected to bioinformatic analysis and revealed the following:Predominantly prevalent strains of PRRSV in Guangdong Province include HP-PRRSV and NADC30-like variants, whereas PCV2 is primarily represented by the 2b and 2d subtypes. Specifically, the amino acid variation patterns exhibited by the PRRSV GP5 and NSP2 proteins of the strains sg_2108, qy_2008, and fs_2108 under environmental selective pressure are remarkably similar to the characteristics of Highly Pathogenic PRRSV; thus, it is inferred that they may possess higher virulence. The detected PCV3 strains were predominantly concentrated within the PCV3a-IM branch. All PRRSV strains involved in this study are wild-type-PRRSV (wt-PRRSV), comprising three recombinant strains and seven highly virulent strains. Among these strains, the *ORF1a* gene exhibited the highest variability in their genomes. Environmental selective pressure may enhance the virulence and immune evasion capabilities of PRRSV and drive mutations in the Cap proteins of PCV2 and PCV3. Conversely, PCV2 and PCV3 strains demonstrated greater stability in genetic evolution. In conclusion, this study enhances the epidemiological data regarding PRRSV, PCV2, and PCV3 in Guangdong Province, China, and is significant for the surveillance, prevention, and active control of these three diseases.

## 1. Introduction

PRRSV is the causative agent of porcine reproductive and respiratory syndrome (PRRS). It is a virus that frequently co-infects with multiple lineage subtype strains and other pathogens, such as ASFV, CSFV, PCV2, and PCV3. These co-infections often exacerbate the pathological damage in infected pigs, increase the diagnostic difficulty of the associated pathogens, and elevate the frequency of PRRSV gene recombination, thereby complicating its control [1]. PRRS primarily results in abortions in sows, mummified or stillborn fetuses, and respiratory disorders, as well as congenital dysplasia in piglets [2]. Among pathogens capable of co-infection with PCV2, PRRSV is the most prevalent. Both PCV2 and PRRSV can cause reproductive disorders and respiratory symptoms, posing challenges for clinical differentiation [3,4]. Due to the extensive phenomenon of genetic recombination and the high frequency of gene mutation, coupled with the lack of a correction mechanism during replication, PRRSV continuously generates new isolates and evades the host’s immune defenses. The recent widespread epidemic of PRRSV in China suggests alterations in its genetic characteristics [5,6].

PRRSV 1 subtypes, with Lelystad as the prototype strain, can be subdivided into three branches, known as the European-like strains. PRRSV 2 subtypes are divided into four lineages: 1, 3, 5, and 8, with VR-2332 as the prototype strain. The strains of PRRSV2 are currently the most pathogenic and widely prevalent types in China, referred to as North American-like strains [7,8]. The high mutation frequency and extensive recombination of PRRSV2 are significant factors contributing to its widespread prevalence, rapid evolution, and the increasing challenges in prevention and control [5,9]. Currently, the primary measures employed in China for controlling PRRS involve the use of modified live virus (MLV) vaccines and inactivated vaccines (IV). However, MLV vaccines exhibit relatively weak cross-protective capabilities and carry risks of viral recombination and mutation, which may lead to antibody-dependent enhancement (ADE). In contrast, the immunological protection conferred by inactivated vaccines is suboptimal. Therefore, there remains a pressing need for the development of safer and more effective PRRS vaccines [10,11,12].

PRRSV2 gained widespread prevalence in China following its initial detection in 1996 [8,13,14]. By 2006, the emergence of HP-PRRSV in lineage 8.7 resulted in substantial economic losses for Chinese swine farms [15,16]. In 2012, the NADC30-like strain of PRRSV lineage 1.8 was first discovered in China, and thereafter, it rapidly spread and caused sustained and severe impacts on the pig population in China [17,18]. Additionally, in 2017, the first outbreak of the NADC34-like strain in lineage 1.5 was detected in China. Presently, HP-PRRSV and NADC30-like strains are the dominant strains in the transmission cycle within Chinese pig farms. Particularly noteworthy are NADC30-like strains, which have garnered attention due to their high frequency of genetic recombination and mutation [19,20,21,22].

Porcine circovirus (PCV) possesses the smallest known animal DNA virus genome, with PCV genotypes currently classified into PCV1 to PCV4. The strains of the PCV1 subtype are non-pathogenic to pigs; those belonging to the PCV4 genotype were discovered in China in 2019. In contrast, as the two primary pathogens responsible for PCVAD, PCV2 and PCV3 are associated with more severe clinical symptoms, and there is no cross-protection between them. Furthermore, the current infection rate has been increasing annually, which underscores the practical significance of studying their epidemiological and genetic evolution characteristics [23,24]. Particularly, PCV2 as the primary pathogen for PCVAD (porcine circovirus-associated diseases), can induce postweaning multisystemic wasting syndrome (PMWS) and porcine dermatitis nephropathy syndrome (PNDS). They exhibit characteristics such as high infectivity, broad transmission range, and the ability to invade multiple tissue organs [25,26]. PCV2 exhibits high genetic variability, comparable to that of RNA viruses [27]. Among its 11 open reading frames (*ORFs*), *ORF2* encodes a high frequency of mutations in the Cap protein [28,29,30]. This gene contains the virus’ major antigenic determinant and the primary mutation sites, rendering it an ideal target for PCV2 subunit vaccine development, phylogeny studies, and epidemiological investigations [31,32,33,34]. Moreover, PCV3 is a significant pathogen within the porcine circovirus, contributing to morbidity in pigs. In addition to its impact, PCV3 demonstrates substantial amino acid and nucleotide homology among different strains, experiences fewer mutations, and retains significant stability in genetic evolution. Importantly, it contains three open reading frames within a genome of merely 2 kb [28,35,36,37]. The Cap protein of PCV3 is encoded by the *ORF2* gene and encompasses the majority of the limited variant of PCV3, yet it exhibits little similarity to the Cap protein of PCV2. PCV2 was confirmed to exist in China in 2002, and since then, successive epidemics of PCV2a and PCV2b have occurred. Among the PCV2a to PCV2h subtypes, the main prevalent genetic subtypes are now PCV2b and PCV2d. Following the first reported case of PCV3 [25], PCV3 occurrences have been reported in various regions of China many times [38,39]. Currently, PCV3 is predominantly represented by PCV3a and PCV3b [35,36,37,40]. Due to the high degree of sequence similarity among most PCV3 genes, accurate identification of PCV3 genotypes or subtypes remains challenging. PCV3 classification primarily involves two methods: one is based on the full-genome amino acid sequences, while the other relies on variations at the 24th and 27th amino acid positions (A24V and R27K) of the Cap protein. This approach divides PCV3 into three genetic subtypes: PCV3a, PCV3b, and PCV3c, and represents a relatively economical and straightforward method. Nevertheless, certain studies have subdivided the PCV3a branch into two stable branches (PCV3a-1 and PCV3a-2) and an intermediate branch (PCV3a-IM) [41].

The isolation and culture of viruses in cells are considered one of the most reliable methods for diagnosing viral diseases. Porcine alveolar macrophages (PAMs) or African green monkey kidney cells (MA-104) and their derived cells (Marc-145) are commonly used for the isolation and identification of PRRSV, while the most frequently used laboratory culture for PCV2 is the porcine kidney cell line (PK-15), which is free of PCV1 contamination. PRRSV can cause Cytopathic effects (CPEs), but these are often not observable until 2 to 5 days; PCV2 does not induce CPE in cells and requires further identification through transmission electron microscopy or immunofluorescence analysis techniques. Currently, the isolation and cultivation of PCV3 are quite challenging, with relatively few successful cases of isolating PCV3 strains. We were unable to achieve success in our attempts to isolate PCV3. Therefore, we only amplified and sequenced the genome sequences of PCV3 (*ORF1* + *ORF2*) from positive samples [42,43].

The GP5 and Nsp2 proteins of PRRSV are encoded by the *ORF5* and *ORF1a* genes, respectively. Both proteins exhibit high variability. Similarly, the Cap protein of PCV2 and PCV3 plays important roles in virus infection, replication, and immune recognition [34,44]. The genetic variability of the aforementioned four proteins is relatively high. These proteins are used as important target proteins for genetic evolution analysis and molecular epidemiological studies of the three viruses mentioned above. To prevent and control PCVAD, various types of commercial vaccines are currently in use, including live attenuated chimeric vaccines, inactivated vaccines, synthetic peptide vaccines, nucleic acid vaccines, subunit vaccines, multivalent vaccines, and viral vector vaccines. However, these vaccines also have drawbacks, such as weak cross-protection and difficulties in the cultivation of the PCV3, making the production of inactivated vaccines challenging. Therefore, there is a continued need for the development of more efficient and safer vaccines [24,45].

Natural selection is one of the five genetic forces (mutation, recombination, selection, gene flow, drift). Environmental positive selection pressure is one of the important reasons for viruses to enhance their environmental adaptability and the main driving force for evolution, and it is also an indispensable and important content in evolutionary analysis [46,47]. Proteins closely associated with viral life activities undergo glycosylation modifications after translation, which not only profoundly affect their own immunogenicity and spatial structure, but also influence various life processes of viruses such as reproduction, infection, and immune evasion. The 137th amino acid residue in the GP5 protein of PRRSV vaccine strains and wild strains is represented by A (Ala) and S (Ser), respectively. Therefore, the 137th amino acid residue is regarded as a distinguishing site between vaccine strains and wild strains [48,49,50]. The 13th and 151st amino acid positions of the PRRSV GP5 protein are considered amino acid sites related to virus virulence. When both the 13th and 151st amino acid positions are R (Arg), it can be classified as a virulent strain [50,51,52]. Furthermore, protein glycosylation post-translation significantly influences its immunogenicity, spatial structure, and diverse biological functions. Most proteins exist in only one type of glycosylation. N-glycosylation of proteins is the most studied and important among the several types of glycosylation modifications [53]. Although the Cap proteins of isolated strains of PCV2 and PCV3 have potential N-glycosylation sites, they often lack glycosylation modifications due to the absence of signal peptides. Previous studies have suggested the existence of three primary glycosylation sites (N34, N44, and N51) in the GP5 protein of PRRSV. Among these, the N44 is the most essential for the recovery of an infectious virus. If glycosylation is absent at residues 34 and 51 of the GP5 protein’s extracellular domain, and viable PRRSV mutants are generated, the sensitivity of these mutants to neutralization by antibodies, as well as the immunogenicity of adjacent neutralizing epitopes, will be enhanced [54].

Individually, the three pathogens mentioned above can inflict severe pathological damage. In practical settings, mixed infections among them are common, compromising the pig’s defenses and significantly heightening susceptibility to infestation by other viruses [55,56]. Guangdong Province serves as a pivotal pork output base in China. As of 2021, reports indicate that approximately 33.37 million pigs flowed into the market, with 20.75 million pigs stocked within the province. In recent years, cases of PRRSV, PCV2, and PCV3, both single and mixed infections, have been continuously reported in the central and northern regions of Guangdong Province. However, research on their epidemiological status remains limited. Therefore, continuous monitoring and analysis of their occurrence and infection status in relevant regions is of considerable practical importance. This study aims to address the aforementioned issues by revealing the genetic variation characteristics and primary epidemic trends of these three viruses, providing reference insights for their prevention and control efforts. To comprehend the current epidemiology and evolutionary trends of PRRSV, PCV2, and PCV3 in North-Central Guangdong Province between 2020 and 2022, samples suspected of being infected with these viruses were collected from multiple pig farms across seven relevant districts, followed by testing. Based on the acquired sequence information, the analysis focused on genetic evolution and the viruses’ environmental adaptability.

## 2. Materials and Methods

### 2.1. Sample Collection and Processing

To ensure that the sample collection was as representative as possible, we conducted sampling across multiple pig farms in seven districts—Guangzhou, Yunfu, Qingyuan, Heyuan, Zhaoqing, Shaoguan, and Foshan—based on the incidence of suspected PRRSV and PCV infections in Guangdong Province, particularly in its northern and central regions, from 2020 to 2022. To prevent cross-contamination between samples, each sample was individually sealed before storage. During sample handling, we adhered strictly to aseptic operation principles, either processing them in batches or in separate laboratories.

Under aseptic conditions, each tissue sample was appropriately sized and mixed with sterile phosphate buffered saline (PBS) to form a tissue homogenate. The homogenate underwent centrifugation at 10,000 rpm for 10 min at 4 °C, after which the supernatant was aspirated to extract total RNA and DNA simultaneously. Subsequently, RT-PCR and PCR assays were performed using primers listed in Table S1 (we have summarized all relevant data and results in Supplementary Materials).

Primers were designed and synthesized utilizing various representative reference strains of PRRSV, PCV2, and PCV3 subtypes to amplify the complete genomes of PRRSV, the *ORF5* gene, and the full genes of PCV2 and PCV3 (Tables S2 and S3). Subsequently, the PCR-amplified products underwent 1% agarose gel electrophoresis, and bands of the expected size were selected and purified. The PCR amplification products were purified and subsequently ligated using the DNA A-Tailing Kit (Takara, Dalian, China) along with pMD18-T vectors (Takara, Dalian, China). Finally, all plasmids were sent to Shanghai Sangon for Sanger sequencing.

### 2.2. Virus Isolation and Nucleic Acid Extraction

Using the same described in Section 2.1, the supernatant of tissue homogenates from PRRSV- and PCV2-positive samples was filtered through a 0.22 μm filter and subsequently inoculated into Marc-145, PAMs, and PK-15 cells that exhibited optimal growth conditions, with a confluence rate of 80% to 100%. The cells were incubated at 37 °C and 5% CO_2_ for two hours. Subsequently, the medium was replaced with RPMI 1640 or DMEM medium containing 2% heat-inactivated Fetal Bovine Serum (FBS) and 1% penicillin-streptomycin for maintenance culture. Following this method, the virus was passaged in cells for three generations, with the virus liquid collected after three freeze thaw cycles. We detected the presence of PRRSV and PCV using RT-PCR or PCR methods. Subsequently, we inoculated the viral solution into the corresponding cells and conducted an indirect immunofluorescence assay. The primary antibody was pig-derived PRRSV/PCV2 positive serum diluted 1:400, and the secondary antibody was Goat Anti-Porcine IgG (H+L)-FITC (SouthernBiotech, Birmingham, AL, USA) diluted 1:1000.

We regularly prepared PAMs cells to ensure high efficiency in virus isolation. Briefly, the piglets were euthanized in accordance with animal welfare guidelines and management practices. After the trachea was ligated and the lungs were completely excised, they were thoroughly cleaned and transferred to a laminar flow hood. Sufficient 1640 medium (containing 2% penicillin-streptomycin) was then injected into the lungs via the trachea. The solution was gently patted and rubbed for 1–2 min, then poured out. This process was repeated two to three times, after which all lavage fluid was collected and centrifuged at 4 °C at 1000 rpm for 10 min. The supernatant was discarded, and the precipitate was resuspended in an appropriate volume of red blood cell lysate and allowed to rest for 3–5 min. After washing with sterile PBS, the cell suspension was filtered through a 70 µm pore diameter cell strainer to obtain PAM cells. The cells were resuspended in 1640 medium (containing 10% heat-inactivated fetal bovine serum) for precipitation, and a suitable quantity of cells was utilized for culture and virus isolation. The excess cells were treated with dimethyl sulfoxide (DMSO) and serum in a defined ratio, and subsequently cooled and stored in liquid nitrogen.

### 2.3. Genetic Evolution and Bioinformatics Analysis

We downloaded the gene sequences of representative reference strains of various lineages and genotypes of PRRSV, PCV2, and PCV3 from the NCBI database (https://www.ncbi.nlm.nih.gov/, accessed on 6 February 2023) for various bioinformatics analyses of the isolated strains, and filtered them using Phylosuite v1.2.2 software [57]. The main information of the reference strains has been listed in Tables S4–S6.

#### 2.3.1. Phylogenetic and Amino Acid Variation Analysis

The NJ (Neighbor-joining) method implemented in MEGA 7 software was employed, and 1000 replicated sampling tests were conducted using Bootstrap to construct genetic evolution trees. These trees were based on the whole genes of the isolates, the *ORF2* genes of PCV2 and PCV3 isolates, and the *ORF5* genes of PRRSV, respectively. Subsequently, the genetic evolution of the isolate variant strains was analyzed based on the results obtained. Furthermore, specific amino acid variants in these crucial proteins (GP5, NSP2, and Cap proteins) of the isolates were identified through comparative analysis of their amino acid sequences with those of representative strains of GP5, NSP2, and Cap proteins for PRRSV, PCV2, and PCV3, respectively.

#### 2.3.2. Genetic Recombination and Sequence Homology Analysis

We examined the genetic variation and sequence similarity among PRRSV, PCV2, and PCV3 isolates by comparing their whole genome sequences with representative reference strains of each genotypic subtype. Nucleotide and amino acid sequences were analyzed using SDT software and MegAlign comparison. Sequence homology heat maps were generated using SDT v1.2. Recombination analyses for the whole genome sequences of PRRSV variants were conducted using RDP 4.0 software, and the results were verified using SimPlot 3.5.1 to determine the specifics of genetic recombination among different strains. Those strains that met the criteria for identification as recombinant through five or more of these methods (*p* < 1 × 10^−6^) were classified as recombinant strains.

#### 2.3.3. Analysis of Natural Selection Pressures and N-Glycosylation Sites

To analyze the role of environmental selection pressure in the genetic evolution of different isolates, forward selection analysis was performed on the GP5 protein of PRRSV isolates and the Cap protein of PCV2 and PCV3 isolates. The gene sequences of each reference strain and isolate were aligned and manually edited using MEGA X. The edited sequences were then imported into Hyphy’s online database, Datamonkey (http://www.datamonkey.org/, accessed on 9 February 2023), and analyzed using Single Likelihood Ancestor Counting (SLAC), Mixed Effects Model of Evolution (MEME) [58], Fixed Effects Likelihood (FEL) [59], and Fast Unconstrained Bayesian Approximation (FUBAR) [60]. Codons were considered under selection if at least three methods identified them. However, in this study, we designated sites identified as positive results by all four methods as positively selected sites [61].

PCV2 and PCV3 Cap proteins lack signal peptides and thus do not undergo glycosylation processes [62,63]. Therefore, we utilized NetNGlyc 1.0 (NetNGlyc 1.0—DTU Health Tech—Bioinformatic Services) to predict and analyze N-glycosylation sites of the GP5 protein from isolated PRRSV strains [64,65].

#### 2.3.4. Analysis and Visualization of Adaptive Evolution Sites

We selected the GP5 protein sequence of the classical representative strain CH-1a of PRRSV genotype 2 lineage 8 (GenBank: AY032626). Two models of the PRRSV GP5 protein were constructed using Alphafold2 (https://alphafold.ebi.ac.uk/, accessed on 10 February 2023) and SWISS-MODEL (SWISS-MODEL (https://www.expasy.org/, accessed on 10 February 2023)). We chose one of the models evaluated as superior by Molprobity (http://molprobity.biochem.duke.edu/, accessed on 12 February 2023). Subsequently, we conducted visual analysis of the selected model using UCSF ChimeraX 1.2.5, showcasing the positive selection sites of PRRSV [66,67,68]. Additionally, for the display analysis of positive selection sites of the PCV2 Cap protein, we selected the existing PDB file 3JCI (2.90 Å) from the PDB database (RCSB PDB: Homepage).

## 3. Results

### 3.1. Sample Detection and Processing

In this study, we collected a total of 226 tissue samples from pigs suspected of being infected with PRRSV and PCV, which died on various farms across seven districts of Guangdong Province from 2020 to 2022. The samples comprised 124 lung tissues, 38 kidney tissues, 24 lymph nodes, 22 spleens, 9 brain tissues, and 9 stillbirths. Among all collected samples, we identified 75 PRRSV-positive samples, 85 PCV2-positive samples, and 17 PCV3-positive samples. This comprised 18 mixed-infection samples of PRRSV and PCV2, 4 mixed-infection samples of PRRSV and PCV3, 10 mixed-infection samples of PCV2 and PCV3, and 4 triple-mixed-infection samples of PRRSV, PCV2, and PCV3. The percentage of mixed-infection with PCV2 in PCV3-positive samples reached 58.8% (10/17). Upon comparing the detection rates in each tissue, it was evident that PRRSV exhibited the highest detection rates in stillbirth, lung, and lymph node tissues; PCV2 was predominantly detected in lymph node and spleen tissues; and PCV3 showed the highest detection rates in lymph node tissues.

**Figure 1:**
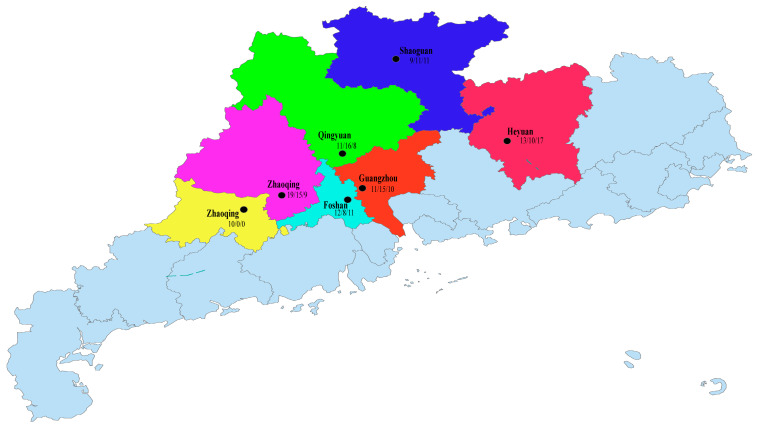
Figure depicting the distribution of sample sources; the three quantities under the name of each district capital represent the number of samples collected by this laboratory in the respective region during the years 2020, 2021, and 2022.

Due to the significant temporal and regional span of the collected samples, we did not record the specific origins and related clinical symptoms of each sample in detail. Fortunately, the corresponding sample data for the sequences obtained in this study are relatively complete. We have provided a detailed presentation and explanation in Figure 1, Table 1, and Supplementary Tables S7–S10.

### 3.2. Virus Isolation, Nucleic Acid Extraction and Sequencing

#### 3.2.1. Nucleic Acid Extraction and Sequencing

Following the methodology outlined in Section 2.2, this study obtained the complete genomic sequences of ten PRRSV strains and twelve PCV2 strains, in addition to the *ORF5* gene sequences of nine additional PRRSV strains. Moreover, we successfully obtained the complete genome sequences (*ORF1* and *ORF2*) of six PCV3 strains. All obtained gene sequences were spliced using SeqMan program in DNASTAR.7.1. They have now been fully uploaded to the NCBI database. The corresponding GenBank accession numbers are listed in Supplementary Material’s Tables S10–S15. The results of agarose gel electrophoresis for the amplification products of the corresponding genes obtained using the Gel System imager are presented in Supplementary Figure S1 and S2.

#### 3.2.2. Isolation of PRRSV and PCV2 Strains

**Figure 2:**
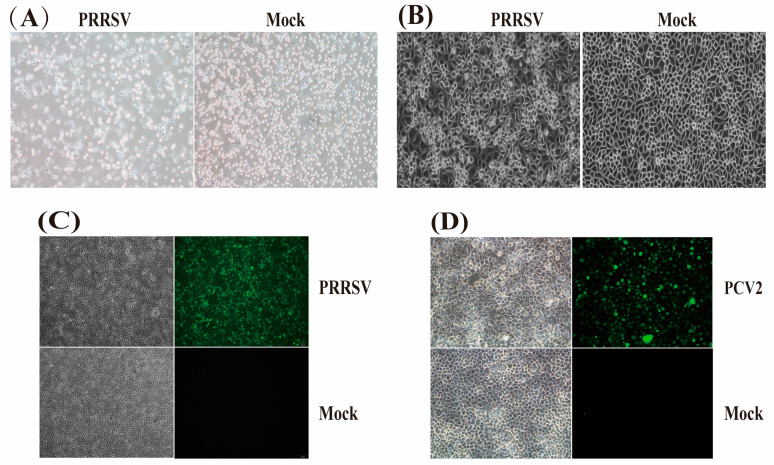
Isolation of PRRSV and PCV2 strains; (**A**) The PRRSV strain was isolated in PAM cells, resulting in significant Cytopathic effects (72 hpi 100×); (**B**) The PRRSV strain was isolated in Marc-145 cells, resulting in significant Cytopathic effects (72 hpi 200×); (**C**) Isolation of PRRSV in Marc-145 cells: observations of lesions in Marc-145 cells post-infection and immunofluorescence assay (IFA) detection using pig-derived PRRSV-positive serum (72 hpi 100×); all isolated PRRSV strains induced similar results; (**D**) Isolation of porcine circovirus type 2 (PCV2) in PK-15 cells: observations of lesions in PK-15 cells post-infection and immunofluorescence assay (IFA) detection using PCV2-positive serum sourced from pigs (72 hpi 200×).

Similarly, following the methods described in Section 2.2, we successfully isolated 10 strains of PRRSV and 12 strains of PCV2 from the corresponding cells; however, we were unable to achieve the isolation of PCV3 in cell cultures (Figure 2).

### 3.3. Genetic Evolution and Bioinformatics Analysis

#### 3.3.1. Phylogenetic Analysis of PRRSV

**Figure 3:**
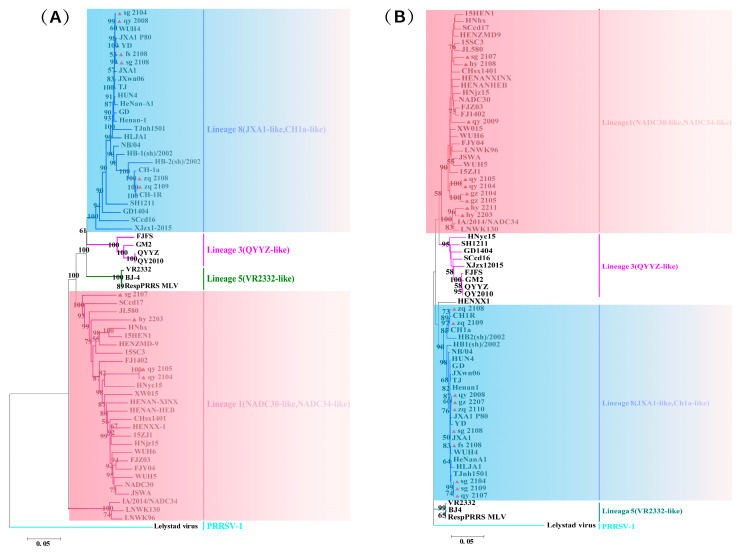
Phylogenetic analysis of PRRSV was conducted, wherein “▲” denotes the sequences of the strains obtained in this study. (**A**) Genetic evolution tree based on the whole genomes of PRRSV isolates and reference strains; (**B**) The phylogenetic tree was constructed based on the *ORF5* gene sequences obtained in this study and the reference strain.

The phylogenetic analysis results based on the full genome and *ORF5* gene of PRRSV are highly consistent (Figure 3): All PRRSV involved in this study belong to genotype 2, with 10 strains classified under lineage 8 and the remaining nine strains belonging to lineage 1. In lineage 8, except for zq_2108 and zq_2109, which belong to the same branch as strain CH-1a, the other eight strains are closely related to the HP-PRRSV strain JXA1 and belong to the JXA1-like genotype. In lineage 1, except for hy_2211, which belong to the same branch as the NADC34-like genotype represented by the strain IA/2014/NADC34 and LNWK130, the other seven strains all belong to the NADC30-like genotype.

The hy_2203 strain occupies different positions in two evolutionary trees, but we categorize it as part of the NADC30-like lineage, or at least as having a closer relationship with NADC30-like strains. This classification is primarily supported by the following reasons: (1) In the genetic evolution tree based on the complete PRRSV genome, the genetic relationship between hy_2203 and NADC30-like strains is closer than with NADC34-like strains; (2) As detailed in the subsequent analysis, hy_2203 is a result of recombination between NADC30-like and NADC34-like strains. Among its three recombination events, the primary parental strain is NADC30, and it has a closer genetic relationship with NADC30 compared to NADC34; (3) hy_2203 exhibits the amino acid deletion pattern we named “111 + 1 + 19”, which is the same as NADC30-like strains.

#### 3.3.2. Phylogenetic Analysis of PCV2

**Figure 4:**
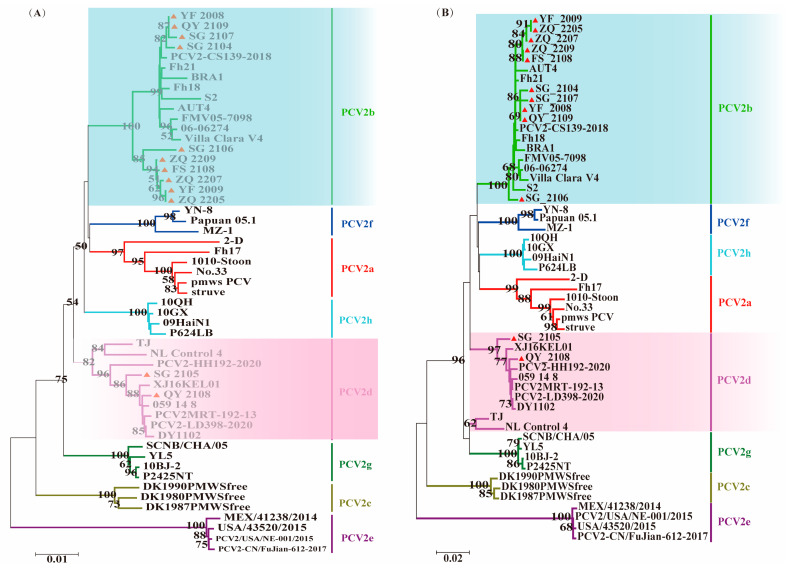
Phylogenetic analysis of PCV2 was conducted, wherein “▲” denotes the sequences of the strains obtained in this study. (**A**) Genetic evolution tree based on the whole genome of PCV2 isolates and reference strains; (**B**) Genetic evolution tree based on the *ORF2* gene of PCV2 isolates and reference strains.

The 12 PCV2 isolated in this study, along with their reference strains, can be classified into eight genotypes, each representing a PCV2 subtype. All 12 isolated strains were found to be distributed within the PCV2b and PCV2d branches, which is consistent with the current predominant epidemic trend of PCV2 in China (Figure 4).

#### 3.3.3. Phylogenetic Analysis of PCV3

**Figure 5:**
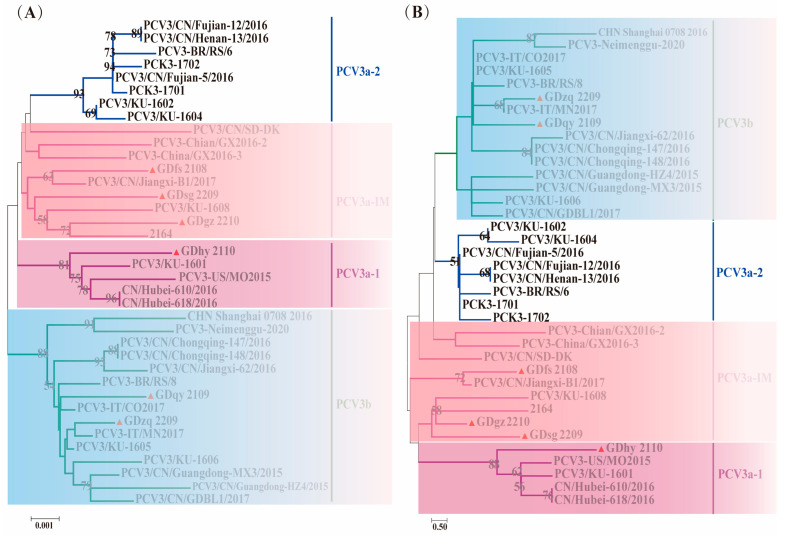
Phylogenetic analysis of PCV3 was conducted, wherein “▲” denotes the sequences of the strains obtained in this study. (**A**) Genetic evolution tree based on the complete coding sequences (*ORF1* + *ORF2*)of PCV3 isolate variant strains and selected reference strains; (**B**) Genetic evolution tree based on the *ORF2* genes of PCV3 isolate variant strains and selected reference strains.

PCV3 genotypes or subtypes cannot be accurately determined at present due to the high degree of sequence similarity of most PCV3 genes. In this study, we adopted the method of subdividing the PCV3a branch into two stable branches (PCV3a-1 and PCV3a-2) and an intermediate branch (PCV3a-IM) for typing [41]. Both genetic evolution trees based on the complete coding sequences of *ORF1* + *ORF2*, *ORF2* of the isolated PCV3 strain and reference strains show consistent results: the isolated PCV3 strain can be classified into two genotypes, 3a and 3b, with distribution observed in subtypes PCV3a-1, PCV3a-IM, and PCV3b, but primarily concentrated in the PCV3a-IM branch (Figure 5).

### 3.4. Amino Acid Variation Analysis of PRRSV

#### 3.4.1. Amino Acid Variation Analysis of GP5 Protein

The 19 *ORF5* nucleotide sequences obtained from this study were translated into amino acid sequences and analyzed, indicating that all corresponding strains are likely wild-type, as the amino acid at position 137 is serine (Ser). The variations of the isolated PRRSV strain GP5 protein in this study mainly concentrate in the signal peptide region, hypervariable regions (HVRs), and the primary neutralizing antigenic epitope region (PNE), while other regions are relatively conserved, and all variations are amino acid mutations without insertions or deletions.

**Figure 6:**
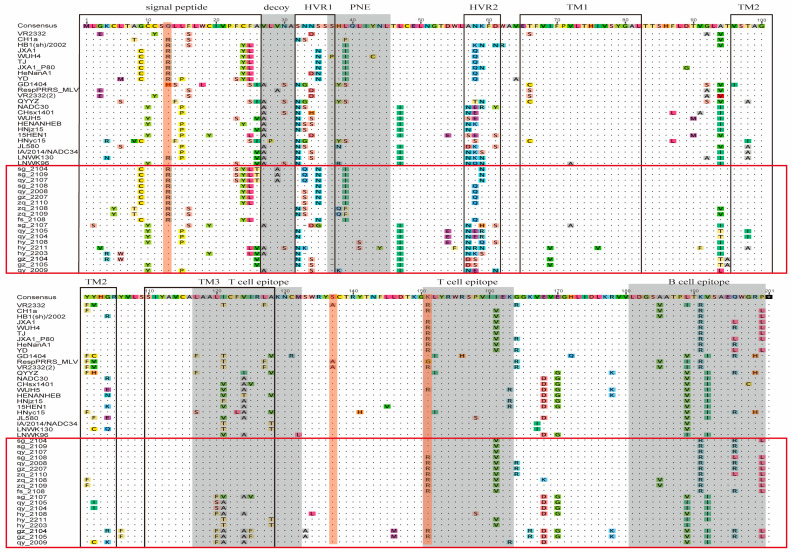
PRRSV GP5 protein amino acid variation analysis, with the red box indicating the sequences of the strains obtained in this study. The black box area from left to right represents the signal peptide region, two hypervariable regions (HVRs), and three transmembrane regions (TMs). The gray area from left to right represents bait epitope region, primary neutralizing antigenic epitope region (PNE), two T-cell epitope regions, and one B-cell epitope region. The orange area indicates distinguishing sites on the GP5 protein for identifying PRRSV vaccine strains, field strains, and variations in virulence.

Strains sg_2108, qy_2008, gz_2207, zq_2110, zq_2108, zq_2109, and fs_2108 exhibit characteristics of highly virulent strains, while other strains may only exhibit moderate to low virulence (Figure 6). The 33rd, 38th, and 39th amino acid residues of the GP5 protein are among five candidate sites closely related to virus neutralization [69]. Several isolated strains have undergone mutations such as N^33^→S^33^/Q^33^/ K^33^ and/or H^38^→Q^38^, which may affect the virus’s role in suppressing the host’s immune response [70]. Strain qy_2009 and 10 other strains have undergone different mutations including H^38^→K^38^ and/or L^39^→F^39^/I^39^, potentially conferring resistance to neutralizing antibodies based on the VR2332 strain.

#### 3.4.2. Amino Acid Variation Analysis of NSP2 Protein

**Figure 7:**
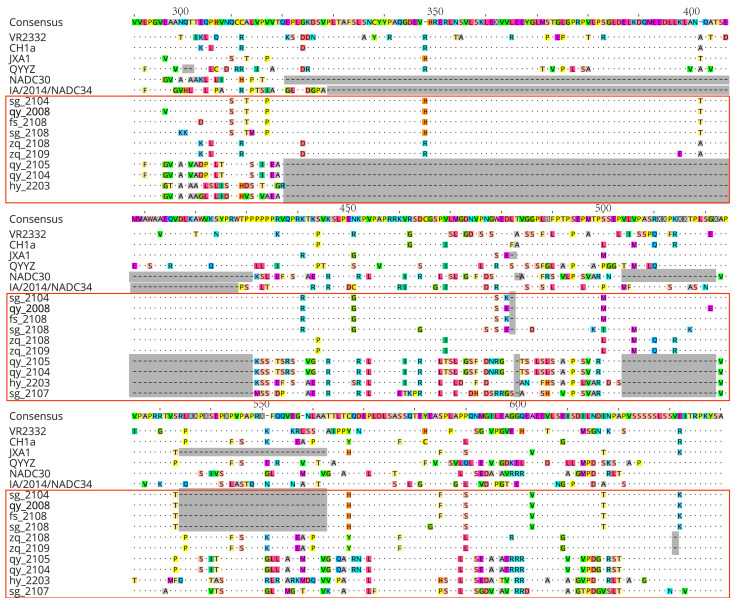
Analysis of amino acid variations in PRRSV Nsp2 protein reveals the presence of the sequences of the strains obtained in this study indicated by the red box, while the gray area denotes segments with amino acid deletions.

Utilizing the amino acid sequences of the NSP2 protein from the prototype strain VR2332 of PRRSV2 and its representative strains as reference standards, we identified 30 discontinuous amino acid deletions at positions 482 and 534–562 in the NSP2 protein of strains sg_2104, qy_2008, fs_2108, and sg_2108. These deletions are consistent with the amino acid deletion pattern observed in the HP-PRRSV strain JXA1 in lineage 8 (“1 + 29”). Consequently, these strains should be classified as JXA1-like strains. Similarly, the Nsp2 protein of qy_2105, qy_2104, hy_2203, and sg_2107 shows discontinuous deletions of 131 amino acids at positions 321-431aa, the 483rd amino acid, and 504-522aa, exhibiting the same amino acid deletion pattern (“111 + 1 + 19”) as NADC30-like in lineage 1 (Figure 7). Therefore, they are likely to belong to the NADC30-like strain, and the aforementioned eight strains may possess similar high pathogenic characteristics to their respective reference strains [71,72].

### 3.5. Amino Acid Variation Analysis of PCV2 and PCV3 Cap Proteins

**Figure 8:**
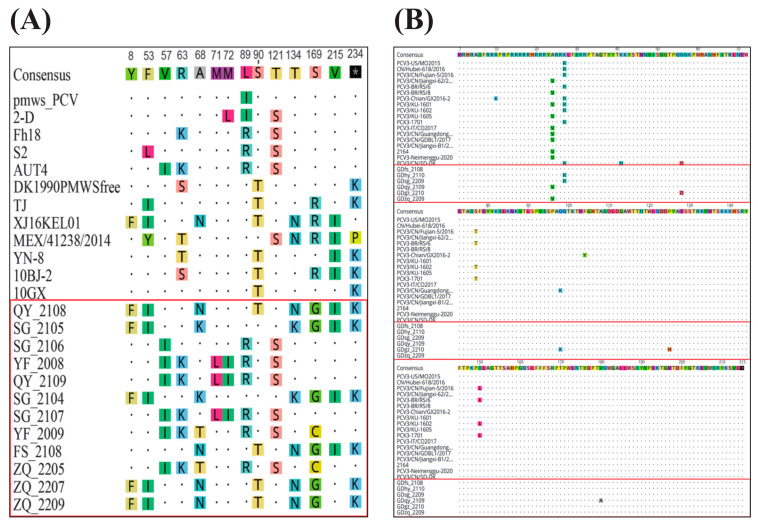
The analysis of amino acid variations in the PCV2 and PCV3 Cap proteins from the sequences of the strains obtained in this study, which are highlighted in red boxes. (**A**) Analysis of amino acid variation in Cap protein of PCV2; (**B**) Analysis of amino acid variation in Cap protein of PCV3.

We found that the similarity of the amino acid sequences of the Cap protein between the isolated strains of PCV2 and the reference strains is comparatively high. PCV3 is more conservative than PCV2, with fewer genetic mutations (Figure 8). Among the isolated PCV2 strains, QY_2108, SG_2105, SG_2104, ZQ_2207, FS_2108, and ZQ_2209 exhibit a high degree of similarity in the sequence of the Cap protein with their reference strains XJ16KEL01 and TJ of the PCV2d subtype. Strains SG_2106, YF_2008, QY_2109, SG_2107, YF_2009, and ZQ_2205, on the other hand, show high similarity in their amino acid sequence with their reference strain AUT4 of the PCV2b subtype. As the only immunogenic protein of PCV2, the amino acid mutations at positions 68, 71, 72, 121, and 134 in the Cap protein of isolated strains are all situated within its antigenic domains (residues 65–87, 113–139) [73]. These mutations affect the virus’s virulence and immunogenicity, particularly the mutation at the 134th amino acid within the second immunogenic epitope (residues 113–139), that may alter the nucleocapsid conformation within this epitope, resulting in a transition from a hydrophobic (contemporary PCV2) to a hydrophilic (archival PCV2) configuration [74,75]. This suggests that the virulence of the six PCV2 isolates with corresponding mutations may be relatively weak. Specifically, strains with amino acid mutations at position 68aa could potentially lead to immune failure [76].

The sequences of the six isolated PCV3 strains are highly conserved compared to the reference strains. Only a few mutations in the Cap protein remain among them. Specifically, compared to the reference strain PCV3/GDBL1/2017, there is only one mutation, V^180^→A^180^, present in two PCV3b isolated strains, namely GDqy_2109. In the PCV3a-IM subtype, compared to the PCV3/CN/Guangdong-HZ4/2015 strain, the GDgz_2210 strain exhibits three point mutations. Conversely, compared to the PCV3-US/MO/2015 strain, the GDfs_2108 strain has only one mutation, R^27^→K^27^. However, no mutations occurred in GDsg_2209 and GDhy_2210 compared to the reference strains. The mutation sites of the PCV3 Cap protein are consistent with previous studies, concentrating on positions 24 and 27. These sites are also an important basis for classifying PCV3 into three subtypes: 3a, 3b, and 3c [41,77,78]. Additionally, mutations occurring in the Cap protein epitope regions A (51-84aa), B (113-132aa), C (161-208aa), and antibody recognition domains (23-35aa and 119-131aa) in some strains may be related to the virus’s virulence and immune evasion. Furthermore, mutations A^24^→V^24^ in strains GDqy-2109 and GDzq-2209, and K^24^→R^24^ in strains GDsg-2209 and GDhy-2110 can further determine their respective genetic subtypes [41,77]. Amino acid substitutions at positions 56 and 98 in the Cap protein of strain GDgz-2210 may potentially alter its immunogenic properties [79].

### 3.6. Genetic Recombination Analysis

**Figure 9:**
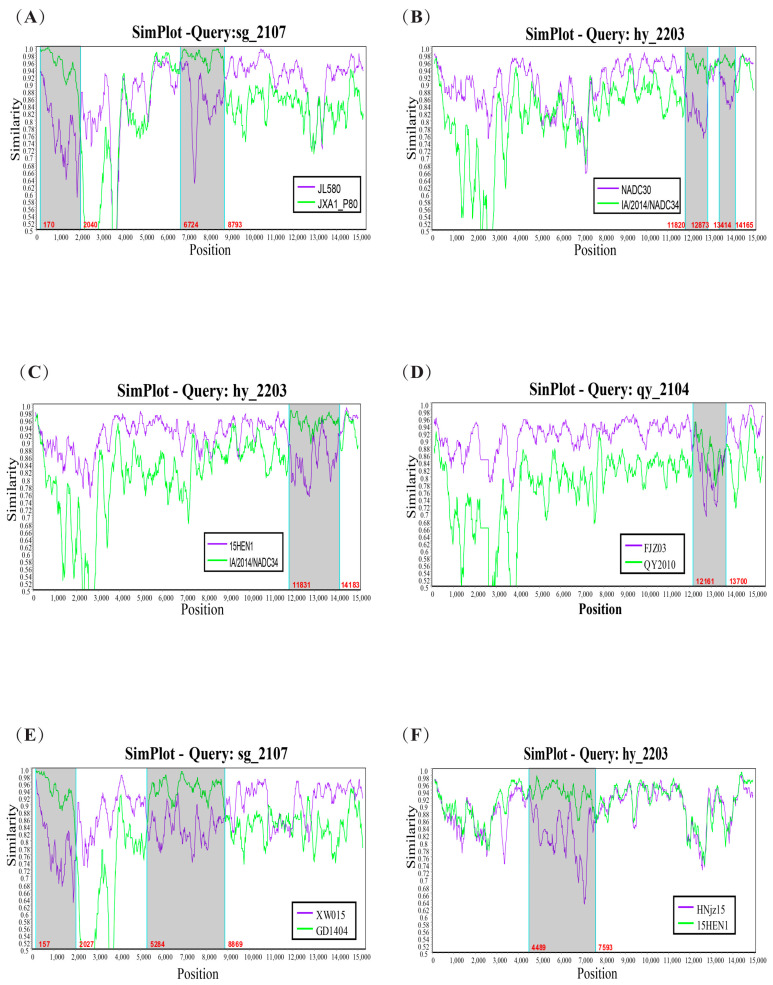
SimPlot recombination analysis graphs of the full genome of the sequences of the PRRSV strains obtained in this study, where the gray area indicates the position of gene recombination. (**A**–**F**): SimPlot recombination analysis graphs corresponding to recombination events 1, 2, 3, 4, 5, and 8 in Table S16.

Preliminary recombination analysis of isolated strains of three viruses showed that no gene recombination was detected in PCV2 and PCV3 isolates, but eight recombination events and three recombinant strains were found in the PRRSV isolates (Table S16 in Supplementary Materials). Through further analysis in SimPlot 3.5.1 software, this study identified six recombination events as well as three recombinant strains: sg_2107, hy_2203, and qy_2104. The *ORF1a* and *ORF4* genes of the isolated strains were the main regions where recombination events occurred (Figure 9).

### 3.7. Sequence Similarity Analysis of Viruses

#### 3.7.1. Sequence Similarity Analysis of PRRSV Whole Genome

Comparisons and analyses were conducted between representative strains of each lineage of PRRSV2 and the nucleotide sequences of isolated strains’ complete genomes. The detailed results are shown in Table 2: the similarity between the complete genome sequences of isolated strains and the reference strains in lineage 1 is the highest, while the similarity with the reference strains in lineage 3 is the lowest. Within the genome of isolated strains, the *ORF1a* gene exhibits the highest variability, followed by the *ORF3* and *ORF5* genes. The *ORF6* gene shows the highest homology with the sequences of reference strains.

#### 3.7.2. Sequence Similarity Analysis of PCV2 and PCV3

**Figure 10:**
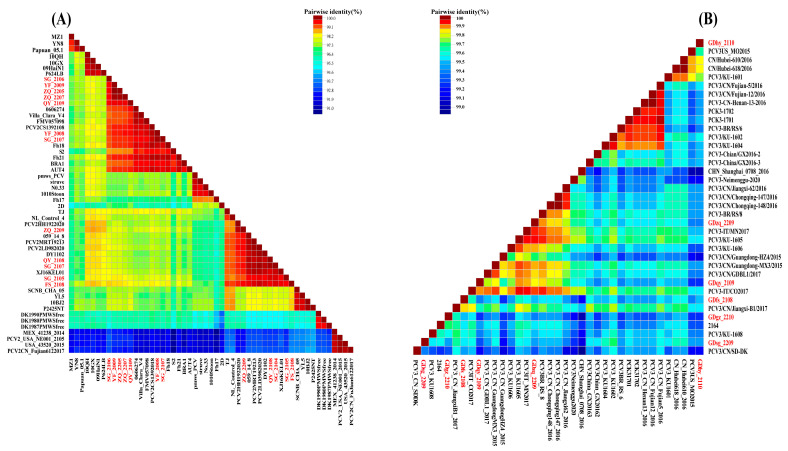
Similarity heat maps of the genome nucleotide sequences of the PCV2 and PCV3 strains obtained in this study, with the isolated strains highlighted in red font; (**A**) Heat map of nucleotide sequence similarity based on the genome sequences of PCV2; (**B**) Heat map of nucleotide sequence similarity based on the genome sequence of PCV3.

The genome sequences of the isolated PCV2 and PCV3 strains and their respective reference strains for each gene subtype were compared and analyzed by SDTv1.2 and MegAlign program in DNASTAR.7.1. The similarity heat map of the full genome sequences is shown in Figure 10. The genome sequences of the PCV2 isolates in this study exhibited a similarity ranging from 96.0% to 100%. The nucleotide similarity between strains SG_2107, YF_2008, QY_2109, YF_2009, ZQ_2205, and SG_2106 and the PCV2b gene subtype reference strains ranged from 97.4% to 99.7%. Strains SG_2105, SG_2104, FS_2108, ZQ_2207, ZQ_2209, and QY_2108 exhibited nucleotide similarity ranging from 95.9% to 99.9% with PCV2d genotype reference strains. The genetic similarity among isolated PCV3 strains ranged from 98.9% to 99.5%. They exhibited the lowest similarity (98.8%) with the PCV3b subtype reference strain and the highest similarity (99.8%) with PCV3a-IM.

### 3.8. N-Glycosylation Site Analysis

This study focuses on the analysis of the N-glycosylation sites of the GP5 protein from detected PRRSV strains. Through predictive analysis, we found that, with varying confidence levels, N34, N44, and N51 N-glycosylation sites were identified in the GP5 protein examined in this research. Among these, glycosylation at N44 is the most conserved, with only 5 out of 19 detected strains exhibiting no related signal. These glycosylation sites primarily influence the virus’s infectivity and may facilitate immune evasion, thereby diminishing the host’s immune defense. In comparison, the reliability of detecting N-glycosylation modifications at the N34 and N51 sites of the strains is either low or absent. This indicates that the glycosylation of the GP5 protein of the PRRSV strains identified in this study has diminished, potentially increasing their sensitivity to antibody neutralization and the immunogenicity of adjacent neutralizing epitopes (specific results are presented in Table S18) [53,80,81,82,83].

### 3.9. Positive Selection Pressure Analysis and Visual Display

**Figure 11:**
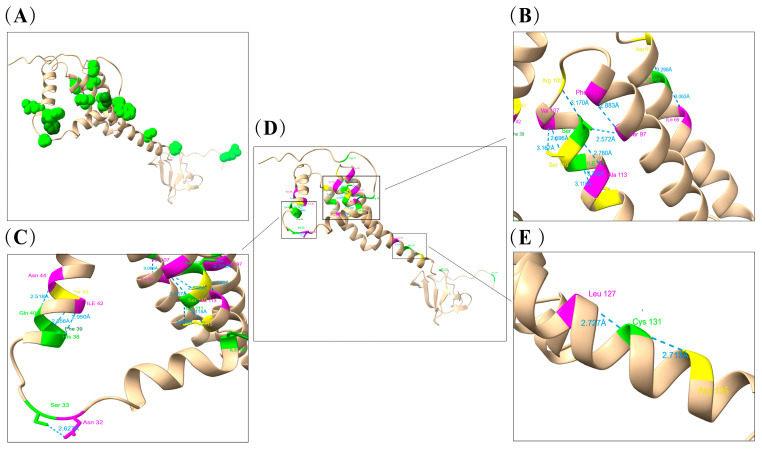
PRRSV GP5 protein model construction and visualization analysis of positively selected sites. The identified sites are presented as light green or dark green spheres. (**A**) Cartoon representation of the PRRSV GP5 protein monomer, site 203 was not displayed; (**B**,**C**,**E**): Microscopic display of positively selected sites and their hydrogen bonds on the GP5 protein model; (**D**) Overall display of the GP5 protein model and its positively selected sites.

**Figure 12:**
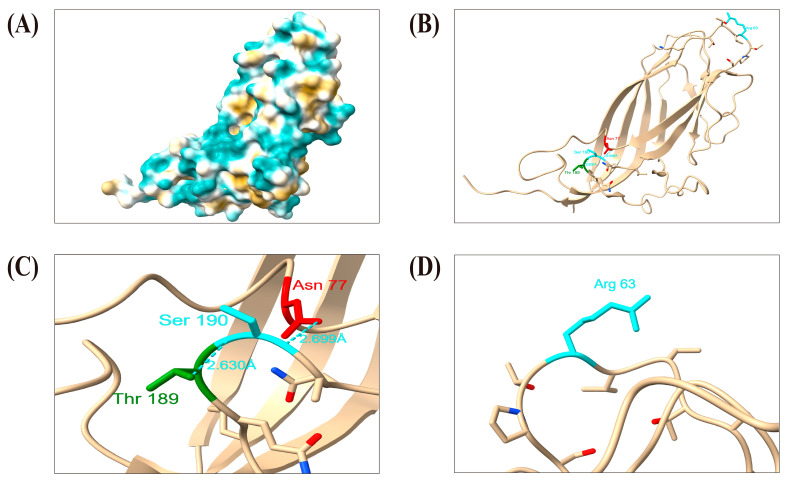
Visualization analysis of the positively selected sites of the PCV2 Cap protein from the isolated strain; (**A**) Displaying the hydrophobic surface of the PCV2 Cap protein; (**B**) Visualizing the positively selected sites of the PCV2 Cap protein through cartoons and Atmos/Bonds display; (**C**,**D**) Analysis of the display of positively selected sites of the PCV2 Cap protein.

The results of the selection pressure analysis in our research are detailed in Tables S19 and S20. The GP5 protein of PRRSV plays a significant role in inducing cross-protection of virus-neutralizing antibodies, as well as their variation. Therefore, studying the mutations it undergoes under selective pressure has become an essential aspect of many molecular evolution studies. The GP5 protein of PRRSV strains contains a total of 15 positively selected sites (11, 18, 19, 20, 33, 38, 39, 40, 65, 109, 111, 131, 158, 199, 203), with residues 38, 39, and 40 located in the primary neutralizing epitope (PNE, 37aa~45aa), while several other sites fall within the common mutation range of the GP5 protein. Environmental selection pressure operates at these sites, possibly accelerating viral evolution and serving as an important factor in the emergence of variations related to “3.4.”. We found that there are N-glycosylation sites at N33 of the GP5 protein in some isolated strains, further confirming the role of immune driving in the emergence of different strains of PRRSV2 [84,85]. We found that the Cap protein of PCV2 in our research has two positive selection sites (63 and 190), while the Cap protein of PCV3 in our research does not have positive selection sites. Additionally, a mutation, R^63^→K^63^, appeared in five isolated PCV2 strains. Therefore, further analysis of the positive selection sites of PRRSV GP5 and PCV Cap proteins is of significant importance (Figure 11 and Figure 12). Apart from amino acids at sites 11, 18–20, 158, and 199, which lack hydrogen bond connections, extensive hydrogen bonds exist among other sites or between their surrounding amino acid residues in the GP5 protein. Most of these sites are located in the primary neutralizing antigenic epitope region (PNE), transmembrane region (TM) and T-cell epitope region of the GP5 protein, playing important roles in the structural stability of the GP5 protein. However, environmental pressures are influence and have already led to mutations in some sites, indicating their potential influence in the genetic evolution of PRRSV and PCV2. Hydrogen bonds play a crucial role in reinforcing the stability of the protein’s secondary structure. Hydrogen bonds are observed between the positively selected site 190(Ser) and 189(Thr), as well as between site 190 (Ser) and 77(Asn) (2.630 Å and 2.699 Å, depicted in cyan). However, no hydrogen bond is detected at site 63 (Arg), where an amino acid mutation, R^63^→K^63^, occurred. In contrast, there is no mutation at position 190aa, indicating significantly higher stability compared to 190aa. Perhaps due to the limited availability of templates related to the PRRSV GP5 protein, the quality of the established models is not particularly high. Among the three models constructed by SWISS-MODEL, although the QMEANDis Co Global value of the most reliable model is displayed as 0.39 ± 0.11, its GMQE value is only 0.09, significantly lower than 1. Among the five models constructed by Alphafold2, the values of “predicted local distance difference test” (PLDDT) range from 70 to 90, indicating their high accuracy and reliability. In the model constructed using AlphaFold2, model 1 exhibits the highest PLDDT value—75.5; In the inspection results of Molprobity, the Ramachandran favored score of model 1 is 94.95%, which is close to 98%, and the Cβ deviation score is reported as 0 (Supplementary Figure S3). Therefore, compared to SWISS-MODEL, the GP5 protein model constructed by Alphafold2 has higher reliability and more accurate results. Hence, we chose the latter for the display analysis of positive selection sites on the PRRSV GP5 protein.

## 4. Discussion

Recently, PRRSV2, PCV2, and PCV3 have been continuously erupting in the southern part of China, spreading rapidly and causing increasingly severe cases of singular or mixed infections, resulting in significant losses to the domestic pig farming industry [86,87]. To understand the latest epidemiology of PRRSV, PCV2, and PCV3 in Guangdong Province from 2020 to 2022, we collected tissue samples from pigs that died from suspected PRRSV and PCV infections across various farms in Guangdong Province. We analyzed these samples for infections caused by PRRSV, PCV2, and PCV3, and isolated and cultured several strains of these viruses in appropriate cells. Subsequent sequence analysis of the isolated strains allowed us to identify the genetic variation patterns and epidemiological characteristics of these three viruses in Guangdong Province. Additionally, we examined the prevalence of mixed infections, thereby addressing the gap in the relevant data.

Before 2011, PRRSV strains reported in China were predominantly of the North American type (PRRSV2). PRRSV-1 was first reported in China after 2011, and it was not until around 2018 that it began to be more frequently detected in Guangdong Province [88,89,90]. By 2006, China reported the initial outbreak of HP-PRRSV and identified a notable feature of 30 amino acid discontinuous deletions in its non-structural protein 2 (NSP2) [16]. Since PCV2 was first reported in China in 1998, its predominant genotypes have undergone two significant shifts. Currently, the major genotypes of circulating strains in China are PCV2b and PCV2d [31]. In the evolutionary and variation processes of PCV2, co-infection among different subtypes can occur, leading to genetic recombination between *ORF1* and *ORF2*, thereby accelerating the emergence of new genotypes. This could be a major factor contributing to the existence of eight genetic subtypes (PCV2a-PCV2h) [28,91,92,93]. PCV3, which was first identified in the United States in 2015, has since become prevalent in China. Its evolutionary rate is significantly lower than that of PCV2, approximately 10^−5^ substitutions/locus/year [94]. PCV3 can be categorized into three genotypes: PCV3a, PCV3b, and PCV3c. Among these, PCV3a can be further divided into three subtypes: PCV3a-1, PCV3a-2, and PCV3a-IM. In recent years, the prevalence of PCV3b strains in China has decreased relative to PCV3a, which aligns with the findings of this study [37,95]. These three viruses frequently exhibit co-infection, either in pairs or all three simultaneously, leading to more severe clinical symptoms and greater economic losses. In this study, we found amino acid variations in the GP5 protein and deletion patterns in the NSP2 protein of PRRSV. Among the PRRSV strains involved in this study, sg_2108, qy_2008, fs_2108, and sg_2104 all exhibited strong virulence characteristics. Notably, the sample from which the sg_2104 sequence was derived was also infected with PCV2, while the sample for the fs_2108 sequence was concurrently infected with PCV2 and PCV3. It can be inferred that co-infection with PCV may be a significant factor contributing to the emergence of virulent PRRSV strains. However, there is currently a lack of recent epidemiological data on their prevalence in Guangdong Province, indicating a need for more comprehensive and in-depth research. We collected 226 samples of suspected PRRSV and PCV infected tissues from deceased pigs in various pig farms within the province. The samples were collected from diverse sources, exhibiting various mixed-infection scenarios, with positive detection rates of PRRSV, PCV2, and PCV3 at 33.2%, 37.6%, and 7.5%, respectively. Our analysis revealed that the predominant circulating strains of PRRSV in Guangdong Province remain HP-PRRSV and NADC30-like strains; PCV2b and PCV2d subtypes have become the most prevalent strain types in Guangdong Province since 2020; PCV3 exhibits relatively high genetic stability, with the majority of circulating strains being PCV3a and PCV3b genetic subtypes. The variant strains isolated in this study belong to the 3a and 3b subtypes. The genetic variation characteristics of their Cap protein are also comparable to those reported in several previous studies [95,96,97,98].

Due to the hydrophobic structure of the PRRSV GP5 protein and the polymorphic nature of the virus particles, it is difficult to purify and crystallize the GP5 protein, and it is also impossible to reconstruct its structure using high-resolution cryo-electron microscopy, so there are currently no available PDB files for it. To enhance research and analysis, we constructed a three-dimensional structural prediction model of the GP5 protein and performed a detailed analysis of the model.

The increase in glycosylation of the PRRSV GP5 protein and the N-glycosylation at certain key sites (N34, N45) can reduce the host’s immune recognition by masking antigenic epitopes and altering the protein’s three-dimensional structure, thereby enhancing the pathogenicity and immune evasion capacity of PRRSV. In the predictive analysis of N-glycosylation modification sites of the GP5 protein sequence, we observed that, in comparison to the reference strain, the degree of N-glycosylation modification in the GP5 protein of the strains obtained in this study increased. Among the 11 potential N-glycosylation modification sites, N44 is highly conserved in the reference sequence. Additionally, the N-glycosylation modifications occurring at position 34aa in the four strains qy_2104, qy_2105, zq_2108, and zq_2109 will directly affect their antigenicity.

In particular, we identified 15 positively selected sites in the GP5 protein and nine of these were situated in critical regions including HVR1, PEN, TM1, TM3, as well as T cell and B cell epitopes. These regions are not only the primary sites of mutations in the GP5 protein, but also are closely linked to its immunogenicity, signal transduction, transmembrane transport, immune recognition, and responses to various biological activities. Moreover, the significant N-glycosylation sites predicted in the GP5 protein are predominantly situated within these critical regions, suggesting that the N-glycosylation modifications occurring at these sites are largely associated with environmental selection pressures. Therefore, PRRSV has evolved a variety of mechanisms for immune evasion and immunosuppression in response to prolonged environmental stress, and the variation in the GP5 protein is closely associated with these environmental factors. Moreover, the widespread genetic recombination of PRRSV makes it more and more difficult to control. Therefore, in the actual production process, we must pay attention to the purification of the breeding environment, avoid mixed infection between different pathogens, and reduce the spread and evolution of pathogens.

Additionally, there are three recombinant strains and six recombination events in the PRRSV isolates and the genetic recombination in both strains occurs in the entire or partial region of the *ORF5* gene. Positions 33aa, 38aa, and 39aa of the GP5 protein are closely associated with viral neutralization and immune evasion mechanisms, and they all represent positive selection sites under selection pressure. Several mutations occurred in multiple PRRSV isolates, including N^33^→S^33^/Q^33^/K^33^, H^38^→Q^38^, H^38^→K^38^, and L^39^→F^39^/I^39^. Similarly, among the 12 isolated PCV2 strains, 5 isolates had mutations at their positive selection sites, such as R^63^→K^63^, indicating that environmental selection pressure is an important driving force for the evolution and variation of these strains.

## 5. Conclusions

In a word, mixed infections of PRRSV, PCV2, and PCV3, either in pairs or all three simultaneously, are commonly observed in pig farms in Guangdong Province. From 2020 to 2022, the predominant prevalent strains of PRRSV in Guangdong Province are still HP-PRRSV and NADC30-like. PCV2 primarily genotypes consist of the PCV2b and PCV2d, while the predominant genotypes of PCV3 strains are PCV3a and PCV3b, with strains within the PCV3a branch mainly concentrated in the PCV3a-IM and PCV3a-1. The prevalence of the 3a subtype strains has been gradually surpassing that of the 3b subtype. The high frequency of gene mutations and widespread recombination phenomena contribute to the continuous emergence of new PRRSV strains, increasing the difficulty of prevention and control. In contrast, the PCV2 and PCV3 strains isolated in this study exhibit higher genetic stability, with fewer gene mutations and no occurrence of genetic recombination. Environmental selection pressure is an important factor in the genetic variation of PRRSV and PCV2, possibly contributing to the emergence of environmentally adaptive variants.

## Figures and Tables

**Table 1:** Positive rate of PRRSV, PCV2, and PCV3 and their co-infection rate in different cities in Guangdong Province from 2020 to 2022.

City	Positive Rate of Samples			PRRSV + PCV2	PRRSV + PCV3	PCV2 + PCV3	PRRSV + PCV3 + PCV3
	PRRSV-Positive Rate	PCV2-Positive Rate	PCV3-Positive Rate				
Guangzhou	6/75	15/85	4/17	1/18	0	3/10	0
Yunfu	2/75	6/85	0	1/18	0	0	0
Qingyuan	14/75	9/85	1/17	3/18	0	1/10	0
Heyuan	18/75	9/85	3/17	2/18	1/4	2/10	1/4
Zhaoqing	8/75	24/85	3/17	3/18	1/4	1/10	1/4
Shaoguan	11/75	8/85	3/17	3/18	0	0	0
Foshan	16/75	14/85	3/17	5/18	2/4	3/10	2/4
Total	75/75	85/85	17/17	18/18	4/4	10/10	4/4

**Table 2:** Nucleotide and amino acid sequence homology analysis of PRRSV isolates.

Gene Name	Nucleotide/Amino Acid Similarity (%)					
	CH-1a	JXA1	VR2332	QYYZ	NADC30	NADC34
whole-genome	81.3~98.0	80.3~98.6	80.6~91.2	72.3~85.7	78.9~86.8	81.0~84.8
	/	/	/	/	/	/
*ORF1a*	75.6~99.5	77.3~99.7	76.6~89.8	72.3~85.7	76.3~92.4	76.8~80.8
	78.1~99.6	79.2~99.8	78.3~91.2	74.4~86.2	76.5~94.2	75.1~81.1
*ORF1b*	86.8~99.7	86.0~99.6	86.5~92.6	84.6~91.3	87.7~94.2	86.4~89.7
	88.1~99.8	87.3~99.4	86.6~92.3	86.2~92.1	88.1~96.2	89.0~90.8
*ORF2a*	86.6~99.5	85.6~99.5	86.1~94.3	85.6~90.3	85.0~91.4	84.7~95.3
	90.1~99.9	88.8~99.8	89.0~92.4	88.2~92.2	86.6~92.2	87.3~97.2
*ORF2b*	87.4~99.5	87.4~99.5	89.2~93.2	89.2~94.6	88.7~94.1	87.4~95.9
	85.4~98.6	87.3~99.2	88.8~93.1	88.4~93.2	87.4~93.3	86.4~95.0
*ORF3*	82.4~99.1	81.6~99.6	82.6~91.4	82.5~90.8	83.4~90.8	82.9~94.2
	78.3~98.2	76.4~98.3	86.4~89.4	84.2~90.9	87.2~91.6	85.3~95.3
*ORF4*	85.3~99.6	83.8~98.5	86.6~91.4	84.2~95.0	87.4~95.2	86.4~95.5
	80.3~98.9	86.5~99.1	88.4~92.1	85.3~96.3	88.8~97.3	88.3~96.1
*ORF5*	85.8~98.8	83.7~99.7	85.3~91.7	82.8~85.1	86.0~93.4	83.7~99.7
	86.9~98.8	85.1~99.8	88.2~93.2	83.5~86.9	88.3~94.2	84.5~99.8
*ORF6*	86.6~99.6	87.7~99.8	89.4~95.3	88.8~90.9	87.5~97.5	87.1~94.1
	88.3~99.7	88.4~99.8	91.2~94.5	90.2~91.3	88.3~95.8	88.6~96.0
*ORF7*	88.7~99.5	88.2~99.7	88.4~94.1	85.2~89.8	90.6~96.2	89.2~94.1
	89.2~97.3	89.7~99.8	89.7~95.2	88.3~90.7	91.2~97.3	88.6~93.6

## Data Availability

The datasets presented in this study are available in online repositories. The names of the repository/repositories and accession number(s) are listed below: https://www.ncbi.nlm.nih.gov/genbank/, accessed on 20 November 2023; OR785784-OR785801, OR800924-OR800933, OR790915-OR790932, and OR790896-OR790914.

## Supplementary Materials

The following supporting information can be downloaded at: https://www.mdpi.com/article/10.3390/v16111687/s1, Table S1. Identification primers for PRRSV, PCV2, and PCV3; Table S2. PRRSV amplified primer information; Table S3. PCV2 and PCV3 amplify primer information; Table S4. PRRSV reference strain information; Table S5. PCV2 reference strain information; Table S6. PCV3 reference strain information; Table S7. The positive rate of PRRSV, PCV2 and PCV3 from 2020 to 2022; Table S8. Summary of clinical sample test results; Table S9. Number of samples collected in different years and regions; Table S10. The GenBank accession numbers of PCV3 genomic DNA (ORF1+ORF2); Table S11. The GenBank accession numbers of PCV2 genomic DNA; Table S12. The GenBank accession numbers of PRRSV genomic RNA; Table S13. The GenBank accession numbers of PCV2 ORF2 gene; Table S14. The GenBank accession numbers of PCV3 ORF2 gene; Table S15. The GenBank accession numbers of PRRSV ORF5 gene; Table S16. Genetic recombination analysis of PRRSV isolates; Table S17. Nucleotide and amino acid sequence homology analysis of PRRSV isolates; Table S18. N glycosylation sites of GP5 protein of PRRSV isolates; Table S19. Positive selection sites for GP5 protein of PRRSV isolates; Table S20. Positive selection sites for Cap protein of PCV2 isolates; Figure S1. Amplification of target genes for PRRSV; Figure S2. Genome amplification of PCV2 and PCV3; Figure S3. Modeling results of the PRRSV GP5 protein and evaluation of the best model quality.
