# Supplementary material for: Epidemiologic Investigation and Genetic Variation Analysis of PRRSV, PCV2, and PCV3 in Guangdong Province, China from 2020 to 2022
Source: Viruses. 2024 Oct 29;16(11):1687. doi: 10.3390/v16111687 (PMC11598979; doi:10.3390/v16111687)
Supplement: Supplementary file 1 [file viruses-16-01687-s001.zip › Supplementary Material.pdf]

Supplementary Material

1 Supplementary Figures and Tables

1.1 Supplementary Tables

Table S1 Identification primers for PRRSV, PCV2, and PCV3

Primer name	primer sequence	Amplicon Length (bp)
PRRSV-F	AAGCTGTTAAACAGGGAGTGG	443
PRRSV-R	CCAAAGAATACCAGCCCATCA	
PCV2-F	CCATATGAAATAAATTACTGAG	785
PCV2-R	CAGCGCACTTCTTTCGTTTTCAG	
PCV3-F	TTACTTAGAGAACGGACTTGTAACG	627
PCV3-R	AAATGAGACACAGAGCTATATTCAG-	

Table S2 PRRSV amplified primer information

Primer name	Sequence（5’～3’）	Amplification length
ORF5-F	GCTCCTTAGTGGTCGATCATGT	810bp
ORF5-R	CCTTTAGAGCATATATCATCACTGGC	
PRRSV1-1F	ACGTATAGGTGTTGGCTCTAT	2556bp
PRRSV1-1R	GGTGACGGCTTCAGTCGG	

PRRSV1-2F	GTAGTTTGGTTTCATTGGGCGGC	2672bp
PRRSV1-2R	GGGTGACAAGACCGGCAACA	
PRRSV1-3F	TTACCTGGTTGCTTTGTGTGTTTCC	3034bp
PRRSV1-3R	ACAACTCAAACCCGGAGGGC	
PRRSV1-4F	TAAAAGGGGTCCTGAAGAACACAAGG	2660bp
PRRSV1-4R	GCCCATCACGGTGCACGG	
PRRSV1-5F	ACAAAGGGCCCTTGTTGCTA	2773bp
PRRSV1-5R	GTGCGGCATTGAGGGATTTTG	
PRRSV1-6F	TTGCCTCACCCGGCAGGC	2532bp
PRRSV1-6R	TCTCGCCAATTAAATGTTACCCCCA	
PRRSV8-1F	CGTATAGGTGTTGGCTCTATGC	2876bp
PRRSV8-1R	CATCAGCTTGGGGACACGTC	
PRRSV8-2F	AGTCTGTCAAAAGCTTGCCAG	2867bp
PRRSV8-2R	CGCCCTCAAGGAGGGACC	
PRRSV8-3F	GACATTCATCATTACACCAGTGGC	2839bp
PRRSV8-3R	AAAGCCAGAGGGCATGGTTG	
PRRSV8-4F	CCCTTATAAGTTGTACCCTGTTAGG	2741bp

PRRSV8-4R	ACCGTGATTTCTCTGTTGTTTCT	
PRRSV8-5F	CCGAGAGGACGGCGCCATTA	2730bp
PRRSV8-5R	ATGGCTTGCAGGCGAACG	
PRRSV8-6F	CCAACCCGGCAGGCAGCC	2530bp
PRRSV8-6R	GGTTCTCGCCAATTAAACTTTACCC	

ORF5-F/ORF5R：Primers for amplification of the PRRSV ORF5 gene in PRRSV-positive samples;

PRRSV1-1F/PRRSV1-1R——PRRSV1-6F/ PRRSV1-6R and PRRSV8-1F/ PRRSV8-1R——PRRSV8-6F/ PRRSV8-6R：Two sets of primers were specifically designed for the reference strains of PRRSV lineage 1 and lineage 8.

Table S3 PCV2 and PCV3 amplify primer information

Primer name	primer sequence（5’～3’）
P2-F	ATCCACGGAGGCCAGTTCG
P2-R	CCCTTCCTCCGTGGATTGTTCTGTA
P3-F	ATTCCTCTTCGGGTACCAGATCGG
P3-R	CCGAAGAGGAATCTTCGCCAATCTC

P2-F/P2-R：Primers for amplification of the PCV2 geneome in PCV2-positive samples；

P3-F/P3-R：Primers for amplification of the PCV3 geneome in PCV3-positive samples；

Table S4 PRRSV reference strain information

Number	Strain	Source	Date	Genetype	Accession
1	Lelystad virus	Europe	1991	PRRSV-1	M96262
2	NADC30	USA	2012	Lineage 1	JN654459
3	HENAN-HEB	China	2012	Lineage 1	KJ143621
4	JL580	China	2015	Lineage 1	KR706343
5	WUH5	China	2015	Lineage 1	KU523366
6	XW015	USA	2013	Lineage 1	KF724409
7	IA/2014/NADC34	USA	2014	Lineage 1	MF326985
8	CHsx1401	China	2014	Lineage 1	KP861625
9	FJZ03	China	2015	Lineage 1	KP860909
10	HNjz15	China	2015	Lineage 1	KT945017
11	15HEN1	China	2015	Lineage 1	KX815413
12	HENAN-XINX	China	2015	Lineage 1	KF611905

13	FJY04	China	2015	Lineage 1	KP860910
14	HNhX	China	2016	Lineage 1	KX766379
15	FJ1402	China	2016	Lineage 1	KX169191
16	WUH6	China	2016	Lineage 1	KU523367
17	HENZMD-9	China	2016	Lineage 1	KU950374
18	15SC3	China	2017	Lineage 1	KX815428
19	LNWK96	China	2017	Lineage 1	MG860516
20	JSWA	China	2017	Lineage 1	KY373214
21	15ZJ1	China	2017	Lineage 1	KX815432
22	LNWK130	China	2017	Lineage 1	MG913987
23	SCcd17	China	2018	Lineage 1	MG914067
24	QYYZ	China	2011	Lineage 3	JQ308798
25	QY2010	China	2010	Lineage 3	JQ743666
26	HNyc15	China	2016	Lineage 3	KT945018
27	SH1211	China	2014	Lineage 3	KF678434
28	GD1404	China	2017	Lineage 3	MF124329
29	SCcd16	China	2018	Lineage 3	MF196905

30	XJZX1-2015	China	2015	Lineage 3	KX689233
31	FJFS	China	2015	Lineage 3	KP998476
32	GM2	China	2012	Lineage 3	JN662424
33	VR2332	USA	2007	Lineage 5	EF536003
34	BJ-4	China	2000	Lineage 5	AF331831
35	RespPRRS MLV	USA	1994	Lineage 5	AF066183
36	HB-1（sh）/2002	China	2002	Lineage 8	AY150312
37	HB2(sh)/2002	China	2004	Lineage 8	AY262352
38	TJ	China	2006	Lineage 8	EU860248
39	GD	China	2007	Lineage 8	EU109503
40	YD	China	2012	Lineage 8	JF748717
41	HeNan-A1	China	2013	Lineage 8	KJ002451
42	CH-1a	China	1996	Lineage 8	AY032626
43	HENXX-1	China	2016	Lineage 8	KU950372
44	CH1R	China	2016	Lineage 8	EU807840
45	HUN4	China	2016	Lineage 8	EF635006
46	JXwn06	China	2016	Lineage 8	EF641008

47	Henan-1	China	2016	Lineage 8	EU200962
48	TJnh1501	China	2017	Lineage 8	KX510269
49	JXA1	China	2006	Lineage 8	EF112445
50	JXA1 P80	China	2008	Lineage 8	FJ548853
51	WUH4	China	2012	Lineage 8	JQ326271
52	NB/04	China	2004	Lineage 8	FJ536165
53	HLJA1	China	2013	Lineage 8	KT351739

Table S5 PCV2 reference strain information

Number	Strain	Country	Date	Genetype	Accession
1	pmws PCV	Canada	1997	2a	AF027217.1
2	2-D	Canada	1999	2a	AF117753.1
3	Fh17	France	2003	2a	AY322004.1
4	1010-Stoon	Spain	2007	2a	GU049340.1
5	struve	USA	2008	2a	KC618389.1
6	No.33	Japan	2001	2a	AB072301.1
7	PCV2-CS139-2018	China	2018	2b	MZ995480.1
8	Fh18	France	2003	2b	AY321987.1
9	S2	China	2003	2b	AY288133.1
10	06-06274	USA	2006	2b	JQ692110.1
11	FMV05-7098	Canada	2005	2b	DQ220731.1
12	Villa Clara V4	Cuba	2009	2b	FN398025.1
13	AUT4	Austria	2003	2b	AY424404.1
14	Fh21	France	2003	2b	AY322001.1
15	BRA1	Brazil	2006	2b	DQ364650.1

16	DK1990PMWSfree	Denmark	2007	2c	EU148505.1
17	DK1980PMWSfree	Denmark	2007	2c	EU148503.1
18	DK1987PMWSfree	Denmark	2007	2c	EU148504.1
19	TJ	China	2002	2d	AY181946.1
20	NL_Control_4	Netherlands	2003	2d	AY484410.1
21	PCV2-HH192-2020	China	2020	2d	MZ995481.1
22	059_14_8	Brazil	2014	2d	KT819169.1
23	PCV2MRT-192-13	india	2013	2d	KJ729075.1
24	PCV2-LD398-2020	China	2020	2d	MZ995484.1
25	DY1102	China	2012	2d	JX406424.1
26	XJ16KEL01	China	2016	2d	MF326371.1
27	MEX/41238/2014	USA	2014	2e	KT795287.1
28	PCV2/USA/NE-001/2015	USA	2015	2e	KT870146.1
29	USA/43520/2015	USA	2015	2e	KT795289.1
30	PCV2-CN/FuJian-612-2017	China	2017	2e	MF589523.1
31	MZ-1	india	2012	2f	LC004746.1
32	YN-8	China	2009	2f	HM776452.1

33	Papuan 05.1	Indonesia	2013	2f	KT369068.1
34	SCNB/CHA/05	China	2005	2g	FJ998185.1
35	YL5	China	2010	2g	HQ202972.1
36	10BJ-2	China	2010	2g	HQ395060.1
37	P2425NT	Vietnam	2008	2g	JX099786.1
38	10QH	China	2010	2h	HQ395058.1
39	10GX	China	2010	2h	HQ395056.1
40	09HaiN1	China	2010	2h	HQ395030.1
41	P624LB	Vietnam	2009	2h	JX099783.1

Table S6 PCV3 reference strain information

Number	Strain	Country	Date	Accession
1	PCV3-US/MO2015	USA	2015	KX778720
2	PCV3/CN/Fujian-5/2016	China	2016	KY075986
3	PCV3/CN/Fujian-12/2016	China	2016	KY075987

4	PCV3-CN-Henan-13-2016	China	2016	KY075988
5	PCV3/CN/Jiangxi-62/2016	China	2016	KY075989
6	PCV3/CN/Chongqing-147/2016	China	2016	KY075990
7	PCV3/CN/Chongqing-148/2016	China	2016	KY075991
8	CN/Hubei-610/2016	China	2016	KY354038
9	CN/Hubei-618/2016	China	2016	KY354039
10	CHN_Shanghai_0708_2016	China	2016	KY865243
11	PCV3/KU-1601	Korea	2016	KY996337
12	PCV3/KU-1602	Korea	2016	KY996338
13	PCV3/KU-1604	Korea	2016	KY996340
14	PCV3/KU-1605	Korea	2016	KY996341
15	PCV3/KU-1606	Korea	2016	KY996342
16	PCV3-BR/RS/6	Brazil	2016	MF079253
17	PCV3-BR/RS/8	Brazil	2016	MF079254
18	PCV3-Chian/GX2016-2	China	2016	MF155642
19	PCV3-China/GX2016-3	China	2016	MF155643
20	PCV3-IT/CO2017	Italy	2017	MF162298

21	PCV3-IT/MN2017	Italy	2017	MF162299
22	PCV3/CN/GDBL1/2017	China	2017	MF405272
23	PCV3/CN/Guangdong-HZ4/2015	China	2015	MF589103
24	PCV3/CN/Guangdong-MX3/2015	China	2015	MF589104
25	PCK3-1701	South Korea	2017	MF611876
26	PCK3-1702	South Korea	2017	MF611877
27	PCV3/CN/Jiangxi-B1/2017	China	2017	MF589107.1
28	PCV3/KU-1608	South Korea	2016	KY996344.1
29	2164	USA	2016	KX458235.1
30	PCV3/CN/SD-DK	China	2021	MW715784.1
31	PCV3-Neimenggu-2020	China	2020	MZ449242.1

Table S7 The positive rate of PRRSV、PCV2 and PCV3 from 2020 to 2022

Year	Number of sample	PRRSV positive rate	PCV2 positive rate	PCV3 positive rate
2020	85	28/85	43/85	7/85
2021	75	15/75	30/75	4/75
2022	66	32/66	12/66	6/66
Total	226	75/226	85/226	17/226

Table S8 Summary of clinical sample test results

Sample source	PRRSV positive rate	PCV2 positive rate	PCV3 positive rate
Lung	45/124（36.2%）	42/124（33.9%）	5/124（4.0%）
Kidney	10/38（26.3%）	18/38（47.4%）	2/38（5.0%）
Lymph nodes	10/24（41.7%）	12/24（50.0%）	7/24（29.2%）
Spleen	3/22（13.6%）	10/22（45.5%）	3/22（13.6%）
Brain	3/9（33.3%）	1/9（11.1%）	0/9（0.0%）
Stillborn foetus	4/9（44.4%）	2/9（22.2%）	0/9（0.0%）
Aggregate	75/226（33.2%）	85/226（36.3%）	17/226（7.5%）

Table S9 Number of samples collected in different years and regions

Date / City	2020	2021	2022	Total
Guangzhou	11	15	10	**36**
Yunfu	10	0	0	**10**
Qingyuan	11	16	8	**35**
Heyuan	13	10	17	**40**
Zhaoqing	19	15	9	**43**
Shaoguan	9	11	11	**31**
Foshan	12	8	11	**31**
**Total**	**85**	**75**	**66**	**226**

Table S10 The GenBank accession numbers of PCV3 genomic DNA（ORF1+ORF2）

Isolated variant strains	City	Date of sample collection	Accession numbers
**GDfs_2108**	Foshan	21 August 2021	OR785784
**GDhy_2110**	Heyuan	26 October 2021	OR785785
**GDgz_2210**	Guangzhou	3 October 2022	OR785786
**GDsg_2209**	Shaoguan	5 September 2022	OR785787
**GDqy_2109**	Qingyuan	17 September 2021	OR785788
**GDzq_2209**	Zhaoqing	3 September 2022	OR785789

Table S11 The GenBank accession numbers of PCV2 genomic DNA

Isolated variant strains	City	Date of sample collection	Accession numbers
**ZQ_2205**	Zhaoqing	23 May 2022	OR785790
**ZQ_2207**	Zhaoqing	8 July 2022	OR785791
**ZQ_2209**	Zhaoqing	3 September 2022	OR785792
**SG_2104**	Shaoguan	25 April 2021	OR785793
**SG_2105**	Shaoguan	3 May 2021	OR785794
**SG_2106**	Shaoguan	14 June 2021	OR785795

**SG_2107**	Shaoguan	5 July 2021	OR785796
**QY_2109**	Qingyuan	17 September 2021	OR785797
**QY_2108**	Qingyuan	26 August 2021	OR785798
**YF_2009**	Yunfu	2 September 2020	OR785799
**YF_2008**	Yunfu	11 August 2020	OR785800
**FS_2108**	Foshan	21 August 2021	OR785801

Table S12 The GenBank accession numbers of PRRSV genomic RNA

Isolated variant strains	City	Date of sample collection	Accession numbers
**zq_2108**	Zhaoqing	17 August 2021	OR800924
**qy_2105**	Qingyuan	4 May 2021	OR800925
**sg_2104**	Shaoguan	25 April 2021	OR800926
**sg_2108**	Shaoguan	8 August 2021	OR800927
**qy_2008**	Qingyuan	27 August 2020	OR800928

**fs_2108**	Foshan	21 August 2021	OR800929
**qy_2104**	Qingyuan	25 April 2021	OR800930
**zq_2109**	Zhaoqing	8 September 2021	OR800931
**sg_2107**	Shaoguan	5 July 2021	OR800932
**hy_2203**	Heyuan	2 March 2022	OR800933

Table S13 The GenBank accession numbers of PCV2 ORF2 gene

Isolated variant strains	City	Date of sample collection	Accession numbers
**ZQ_2205**	Zhaoqing	23 May 2022	OR790915
**ZQ_2207**	Zhaoqing	8 July 2022	OR790916
**ZQ_2209**	Zhaoqing	3 September 2022	OR790917
**SG_2104**	Shaoguan	25 April 2021	OR790918
**SG_2105**	Shaoguan	3 May 2021	OR790919
**SG_2106**	Shaoguan	14 June 2021	OR790920

**SG_2107**	Shaoguan	5 July 2021	OR790921
**QY_2109**	Qingyuan	17 September 2021	OR790922
**QY_2108**	Qingyuan	26 August 2021	OR790923
**YF_2009**	Yunfu	2 September 2020	OR790924
**YF_2008**	Yunfu	11 August 2020	OR790925
**FS_2108**	Foshan	21 August 2021	OR790926

Table S14 The GenBank accession numbers of PCV3 ORF2 gene

Isolated variant strains	City	Date of sample collection	Accession numbers
**GDfs_2108**	Foshan	21 August 2021	OR790927
**GDhy_2110**	Heyuan	26 October 2021	OR790928
**GDgz_2210**	Guangzhou	3 October 2022	OR790931
**GDsg_2209**	Shaoguan	5 September 2022	OR790929

**GDqy_2109**	Qingyuan	17 September 2021	OR790930
**GDzq_2209**	Zhaoqing	3 September 2022	OR790932

Table S15 The GenBank accession numbers of PRRSV ORF5 gene

Isolated variant strains	Sample source(City)	Date of sample collection	Accession numbers
**fs_2108**	Foshan	21 August 2021	OR790896
**gz_2104**	Guangzhou	15 April 2021	OR790897
**gz_2105**	Guangzhou	15 May 2021	OR790898
**gz_2207**	Guangzhou	14 July 2022	OR790899
**hy_2108**	Heyuan	26 August 2021	OR790900
**hy_2203**	Heyuan	2 March 2022	OR790901
**hy_2211**	Heyuan	7 November 2022	OR790902
**qy_2008**	Qingyuan	27 August 2020	OR790903
**qy_2009**	Qingyuan	3 September 2020	OR790904
**qy_2104**	Qingyuan	25 April 2021	OR790905
**qy_2105**	Qingyuan	4 May 2021	OR790906
**qy_2107**	Qingyuan	4 July 2021	OR790907
**sg_2104**	Shaoguan	25 April 2021	OR790908

**sg_2107**	Shaoguan	5 July 2021	OR790909
**sg_2108**	Shaoguan	8 August 2021	OR790910
**sg_2109**	Shaoguan	11 September 2021	OR790911
**zq_2108**	Zhaoqing	17 August 2021	OR790912
**zq_2109**	Zhaoqing	8 September 2021	OR790913
**zq_2110**	Zhaoqing	12 October 2021	OR790914

Table S16 Genetic recombination analysis of PRRSV isolates

Recombinant event	Recombinant strain	Main parental strain	Main parental strain	Recombination analysis method						
				R	G	B	M	C	S	T
1	sg_2107	JL580	JXA1_P80	+	+	+	+	+	+	+
2	hy_2203	NADC30	IA/2014/NADC34	+	+	+	+	+	+	+
3	hy_2203	15HEN1	IA/2014/NADC34	+	+	—	+	+	+	+
4	qy_2104	FJZ03	QY2010	+	—	+	+	+	+	+
5	sg_2107	XW015	GD1404	+	—	—	+	+	+	+
6	sg_2107	HENAN-HEB	JL580	+	—	—	+	+	—	—
7	qy_2105	TJ	WUH4	—	+	—	—	—	+	—

8	hy_2203	HNjz15	15HEN1	+	—	+	+	+	—	+

The letters R, G, B, M, C, S, and T represent the seven detection methods in RDP4.0 software: RDP, GENECONV, BoostScan, MAXCHI, CHIMAERA, SISCAN, and 3SEQ.

Table S17 Nucleotide and amino acid sequence homology analysis of PRRSV isolates

gene name	Nucleotide/amino acid similarity (%)					
	CH-1a	JXA1	VR2332	QYYZ	NADC30	NADC34
whole-genome	81.3~98.0	80.3~98.6	80.6~91.2	72.3~85.7	78.9~86.8	81.0~84.8
	/	/	/	/	/	/
ORF1a	75.6~99.5	77.3~99.7	76.6~89.8	72.3~85.7	76.3~92.4	76.8~80.8
	78.1~99.6	79.2~99.8	78.3~91.2	74.4~86.2	76.5~94.2	75.1~81.1
ORF1b	86.8~99.7	86.0~99.6	86.5~92.6	84.6~91.3	87.7~94.2	86.4~89.7
	88.1~99.8	87.3~99.4	86.6~92.3	86.2~92.1	88.1~96.2	89.0~90.8
ORF2a	86.6~99.5	85.6~99.5	86.1~94.3	85.6~90.3	85.0~91.4	84.7~95.3
	90.1~99.9	88.8~99.8	89.0~92.4	88.2~92.2	86.6~92.2	87.3~97.2
ORF2b	87.4~99.5	87.4~99.5	89.2~93.2	89.2~94.6	88.7~94.1	87.4~95.9
	85.4~98.6	87.3~99.2	88.8~93.1	88.4~93.2	87.4~93.3	86.4~95.0
ORF3	82.4~99.1	81.6~99.6	82.6~91.4	82.5~90.8	83.4~90.8	82.9~94.2

	78.3~98.2	76.4~98.3	86.4~89.4	84.2~90.9	87.2~91.6	85.3~95.3
ORF4	85.3~99.6	83.8~98.5	86.6~91.4	84.2~95.0	87.4~95.2	86.4~95.5
	80.3~98.9	86.5~99.1	88.4~92.1	85.3~96.3	88.8~97.3	88.3~96.1
ORF5	85.8~98.8	83.7~99.7	85.3~91.7	82.8~85.1	86.0~93.4	83.7~99.7
	86.9~98.8	85.1~99.8	88.2~93.2	83.5~86.9	88.3~94.2	84.5~99.8
ORF6	86.6~99.6	87.7~99.8	89.4~95.3	88.8~90.9	87.5~97.5	87.1~94.1
	88.3~99.7	88.4~99.8	91.2~94.5	90.2~91.3	88.3~95.8	88.6~96.0
ORF7	88.7~99.5	88.2~99.7	88.4~94.1	85.2~89.8	90.6~96.2	89.2~94.1
	89.2~97.3	89.7~99.8	89.7~95.2	88.3~90.7	91.2~97.3	88.6~93.6

Table S18 N glycosylation sites of GP5 protein of PRRSV isolates

Strain	Lineage	N^{30}	N^{32}	N^{33}	N^{34}	N^{35}	N^{43}	N^{44}	N^{50}	N^{51}	N^{56}	N^{57}	NGSs number
Sg_2107	1	√					▲		√				3
hy_2108	1	√		♦	√			▲		√			4
hy_2203	1		√	√			▲		√		√		5
hy_2211	1				♦					√			1
qy_2009	1	√		√				▲		√			4
qy_2104	1			√	▲			▲		√			4
qy_2105	1			♦	▲			▲		√			3
gz_2104	1		√	√			▲		√				4
gz_2105	1		√	√			▲		√				4
IA/2014/NADC34	1		√	√				▲		√		√	5
NADC30	1				▲			▲		√			3
Ch1a	8				▲			▲		√			3

JXA1	8	▲			♦	√		▲		√			4
sg_2104	8	√			√	√		▲		√			5
sg_2109	8	√			√	√		▲		√			5
sg_2108	8	▲			√	√		▲		√			5
qy_2008	8	▲			√	√		▲		√			5
qy_2107	8	√			√	√		▲		√			5
gz_2207	8	▲			√	√		▲		√			5
fs_2108	8	▲			♦	√		▲		√			4
zq_2108	8				▲			▲		√			3
zq_2109	8				▲			▲		√			3
zq_2110	8	▲			√	√		▲		√			5

N-glycosylation sites of GP5 protein of PRRSV isolates. The symbol "▲" Indicates N-glycosylation sites with a "Jury agreement" score of 9/9, while "♦" Indicates sites where glycosylation modification cannot occur; "√" Indicates sites where N-glycosylation may occur, but with low confidence.

Table S19 Positive selection sites for GP5 protein of PRRSV isolates.

Site	FUBAR (Post.Pro)	MEME (*P* value)	FEL (*P* value)	SLAC (*P* value)
11	0.950	0	0.0149	0.0201
18	0.956	0.06	0.0435	0.0887
19	0.932	0.04	0.0262	0.0406
20	0.976	0	0.0057	0.0390
33	0.940	0.04	0.0258	0.0390
38	0.999	0	0.0027	0.00474
39	0.995	0.01	0.0131	0.0135
40	0.982	0.02	0.0160	0.0251
65	0.995	0.01	0.0049	0.0140
109	0.993	0.01	0.0250	0.0493
111	0.963	0	0.0461	0.0103
131	0.982	0.01	0.0065	0.0264
158	0.955	0.01	0.0155	0.0771
199	0.956	0.04	0.0235	0.0736
203	0.998	0	0.0007	0.0094

Table S20 Positive selection sites for Cap protein of PCV2 isolates.

Site	FUBAR (Post.Pro)	MEME (*P* value)	FEL (*P* value)	SLAC (*P* value)
63	0.997	0.01	0.0040	0.0204
190	0.984	0.02	0.0140	0.0402

1.2 Supplementary Figures

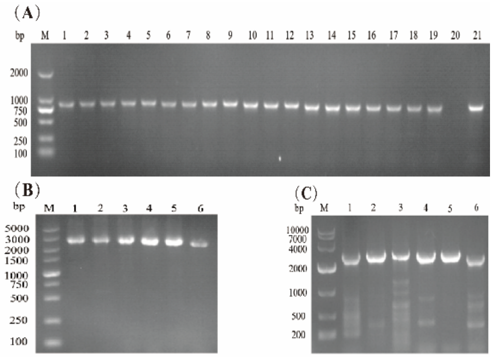

Supplementary Figure S1. Amplification of target genes for PRRSV;（A）1-19 represents the amplification products of the ORF5 gene from 10 isolated PRRSV strains and disease material, while 20 and 21 represent the negative and positive controls, respectively. (The corresponding strains for holes 1-19 are: sg_2104, sg_2109, qy_2107, sg_2108, qy_2008, gz_2207, zq_2110, zq_2108, zq_2109, fs_2108, sg_2107, qy_2105, qy_2104, hy_2108, hy_2211, hy_2203, gz_2104, gz_2105, qy_2009)；(B) Whole genome amplification of the isolate sg_2108 from Lineage 8 of PRRSV, with individual amplified fragments of the whole genome of sg_2108 in wells 1 to 6, respectively; (C) Whole genome amplification of strain hy-2203 isolated from Lineage 1 of PRRSV, wells 1 to 6 are individual amplified fragments of the whole genome of strain hy-2203, respectively.

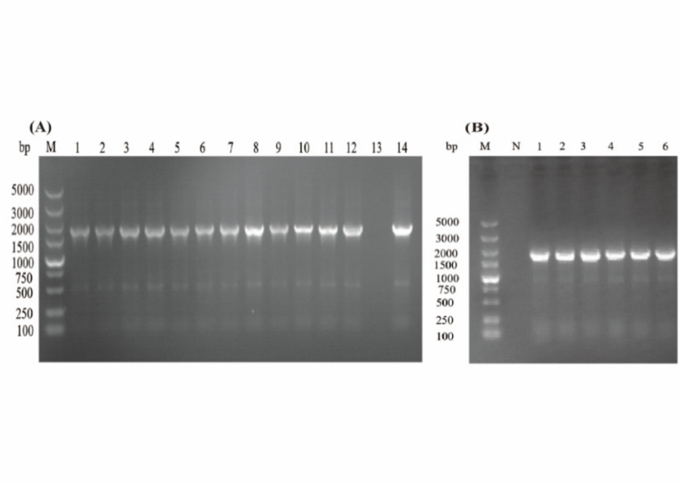

Supplementary Figure S2. Genome amplification of PCV2 and PCV3; (A) QY_2108, SG_2105, SG_2106, YF_2008, QY_2109, SG_2104, SG_2107, YF_2009, ZQ_2205, ZQ_2207, ZQ_2209, and FS_2108）；（B）1-6 are the amplification products of the full genomes of 6 isolated PCV3 strains, "N" is the negative control, and the strains corresponding to wells 1-6 are: GDfs_2108, GDsg_2209, GDgz_2210, GDhy_2110, GDqy_2109, and GDzq_2209.

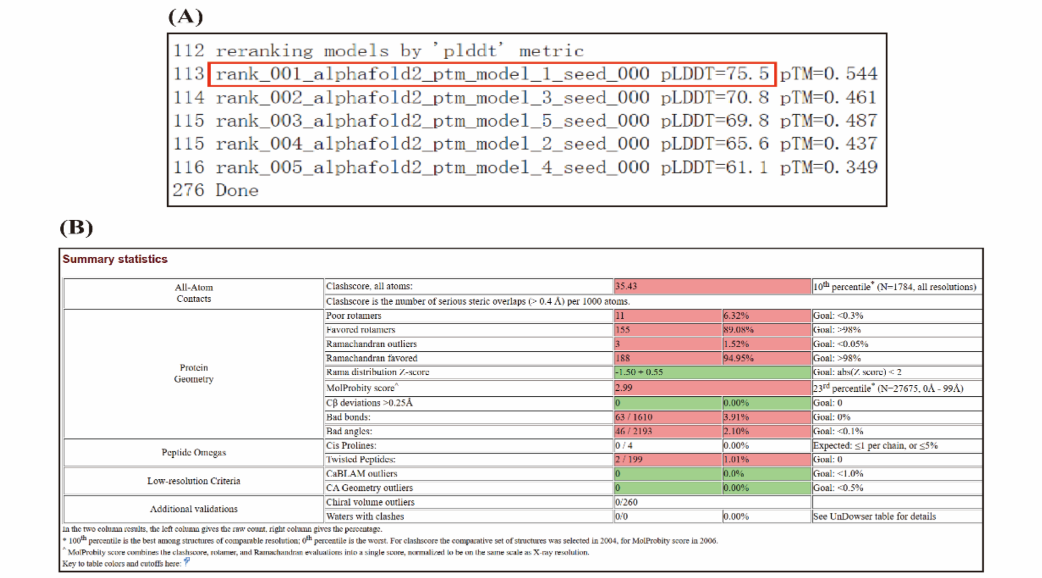

(A)

```
112 reranking models by 'plddt' metric
113 rank_001_alphafold2_ptm_model_1_seed_000 pLDDT=75.5 pTM=0.544
114 rank_002_alphafold2_ptm_model_3_seed_000 pLDDT=70.8 pTM=0.461
115 rank_003_alphafold2_ptm_model_5_seed_000 pLDDT=69.8 pTM=0.487
115 rank_004_alphafold2_ptm_model_2_seed_000 pLDDT=65.6 pTM=0.437
116 rank_005_alphafold2_ptm_model_4_seed_000 pLDDT=61.1 pTM=0.349
276 Done
```

(B)

Summary statistics

All-Atom Contacts	Clashscore, all atoms:	35.43		10th percentile* (N=1784, all resolutions)
	Clashscore is the number of serious steric overlaps (> 0.4 Å) per 1000 atoms.			
Protein Geometry	Poor rotamers	11	6.32%	Goal: <0.3%
	Favored rotamers	155	89.08%	Goal: >98%
	Ramachandran outliers	3	1.52%	Goal: <0.05%
	Ramachandran favored	188	94.95%	Goal: >98%
	Rama distribution Z-score	-1.50 + 0.55		Goal: abs(Z score) < 2
	MolProbity score^	2.99		23rd percentile* (N=27675, 0Å - 99Å)
	Cβ deviations >0.25Å	0	0.00%	Goal: 0
	Bad bonds:	63 / 1610	3.91%	Goal: 0%
	Bad angles:	46 / 2193	2.10%	Goal: <0.1%
Peptide Omegas	Cis Prolines:	0 / 4	0.00%	Expected: ≤1 per chain, or ≤5%
	Twisted Peptides:	2 / 199	1.01%	Goal: 0
Low-resolution Criteria	CaBLAM outliers	0	0.0%	Goal: <1.0%
	CA Geometry outliers	0	0.00%	Goal: <0.5%
Additional validations	Chiral volume outliers	0/260		
	Waters with clashes	0/0	0.00%	See UnDowser table for details

In the two column results, the left column gives the raw count, right column gives the percentage.
* 100th percentile is the best among structures of comparable resolution; 0th percentile is the worst. For clashscore the comparative set of structures was selected in 2004, for MolProbity score in 2006.
^ MolProbity score combines the clashscore, rotamer, and Ramachandran evaluations into a single score, normalized to be on the same scale as X-ray resolution.
Key to table colors and cutoffs here:

Supplementary Figure S3： Modeling results of the PRRSV GP5 protein and evaluation of the best model quality（A）The GP5 protein sequence of the classic PRRSV strain CH-1a was predicted using AlphaFold2, yielding five models with corresponding quality scores. Notably, Model 1, indicated in red, exhibits the highest pLDDT value, signifying superior reliability；（B）The quality assessment results of Model 1 on the Molprobity platform, indicating that the model is relatively reliable.

The figure legends of full-length gels and blots

（For clarity and annotation purposes, each figure is presented here in duplicate: one displaying the original image and the other serving as a guide for annotations）

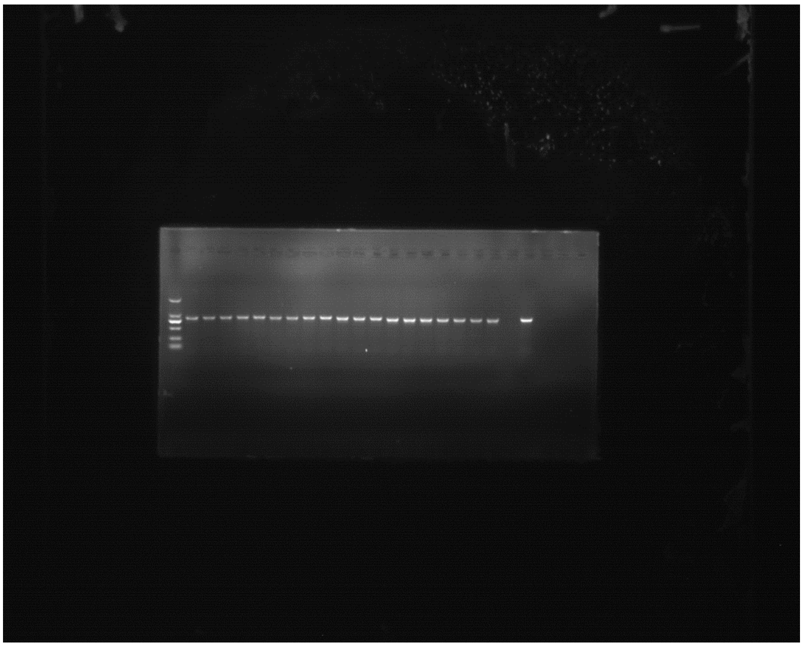

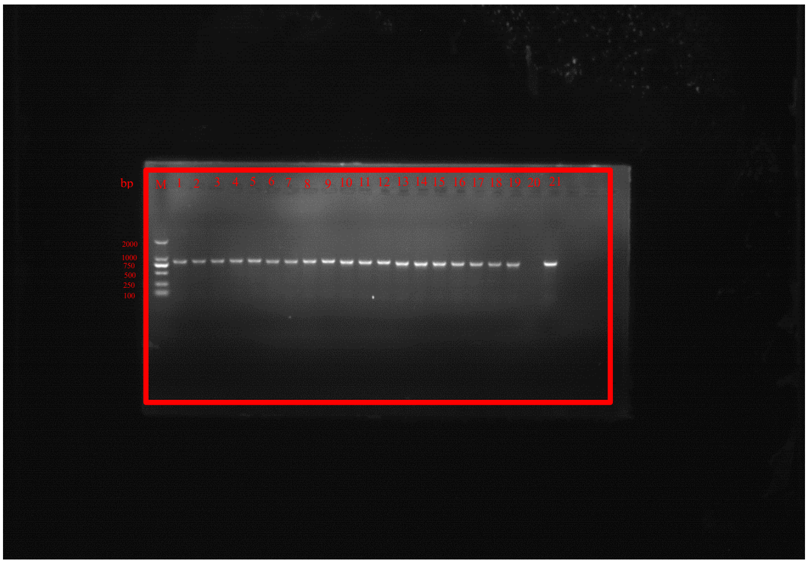

The original nucleic acid gel image corresponding to Supplementary Figure S1 (A); Supplementary Figure S2 (A) depicts a cropped section of this image, encompassing wells 1 to 22 (as indicated by the red box). The order of sample addition, marker band sizes, and all

graphic annotations remain consistent with those in the manuscript, and have been highlighted in red text.

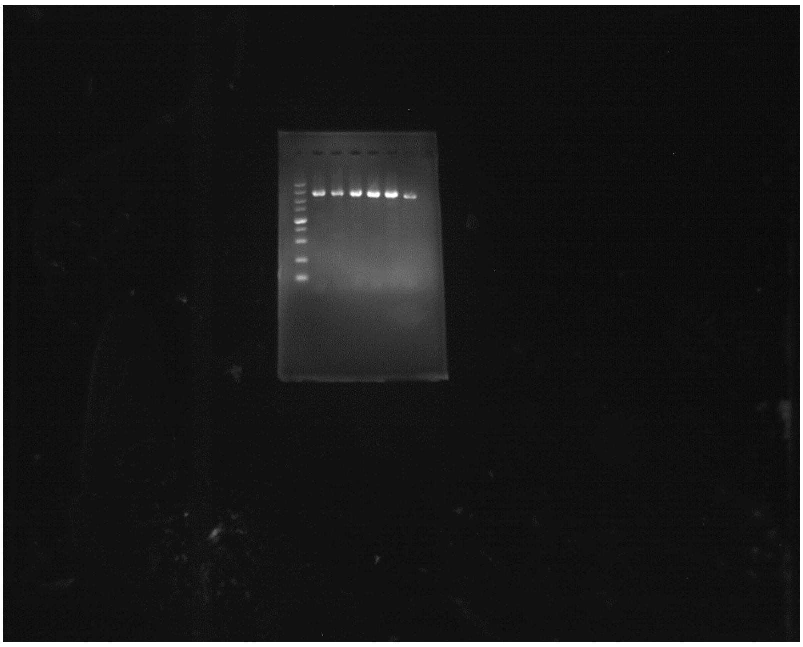

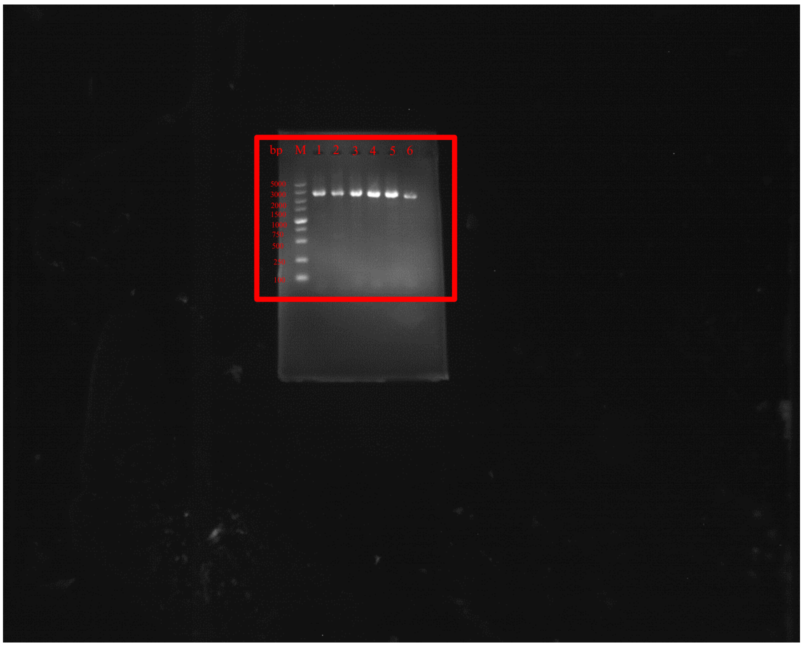

The original nucleic acid gel image corresponding to Supplementary Figure S3 (B); Supplementary Figure S4 (B) depicts a cropped section of this image, encompassing wells 1 to 7 (as indicated by the red box). The order of sample addition, marker band sizes, and all graphic

annotations remain consistent with those in the manuscript, and have been highlighted in red text.

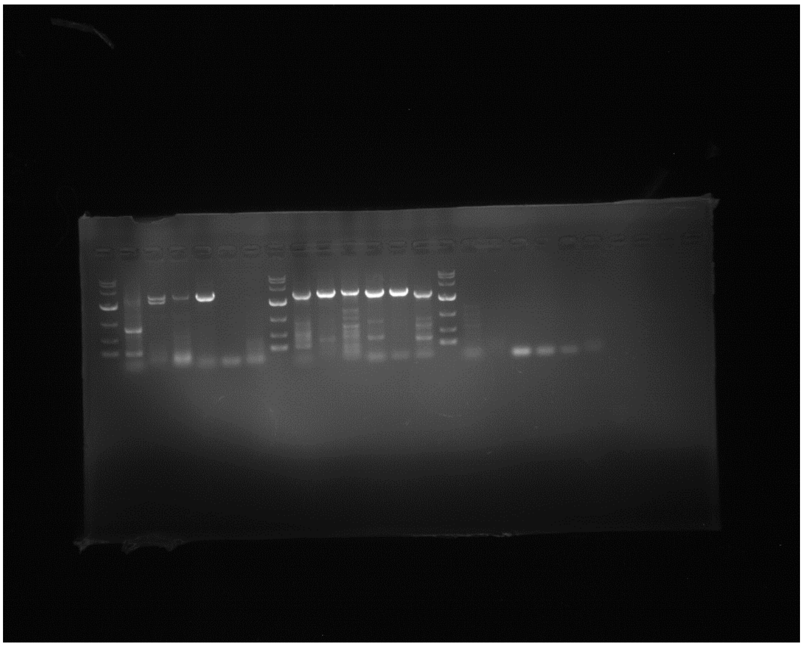

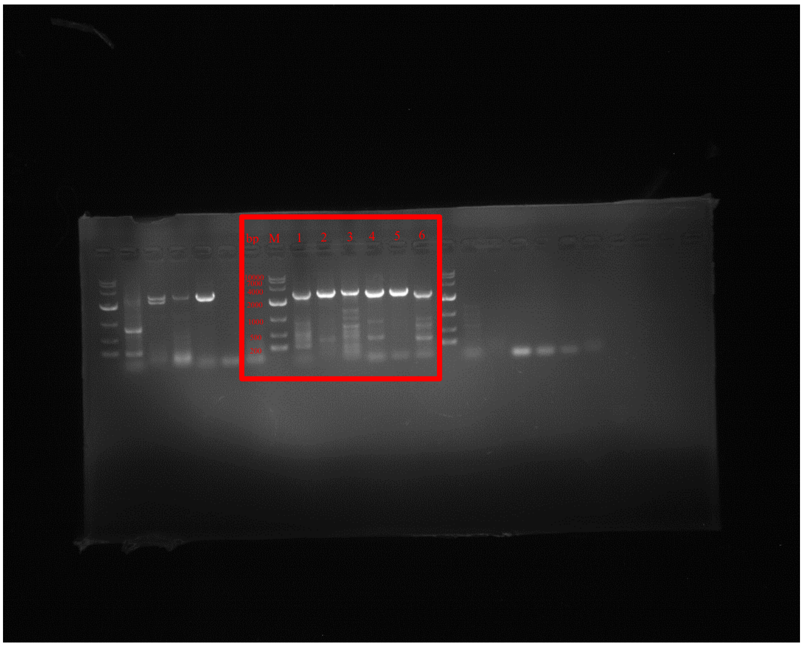

The original nucleic acid gel image corresponding to Supplementary Figure S5 (C); Supplementary Figure S6 (C) depicts a cropped section of this image, encompassing wells 8 to 14 (as indicated by the red box). The order of sample addition, marker band sizes, and all

graphic annotations remain consistent with those in the manuscript, and have been highlighted in red text.

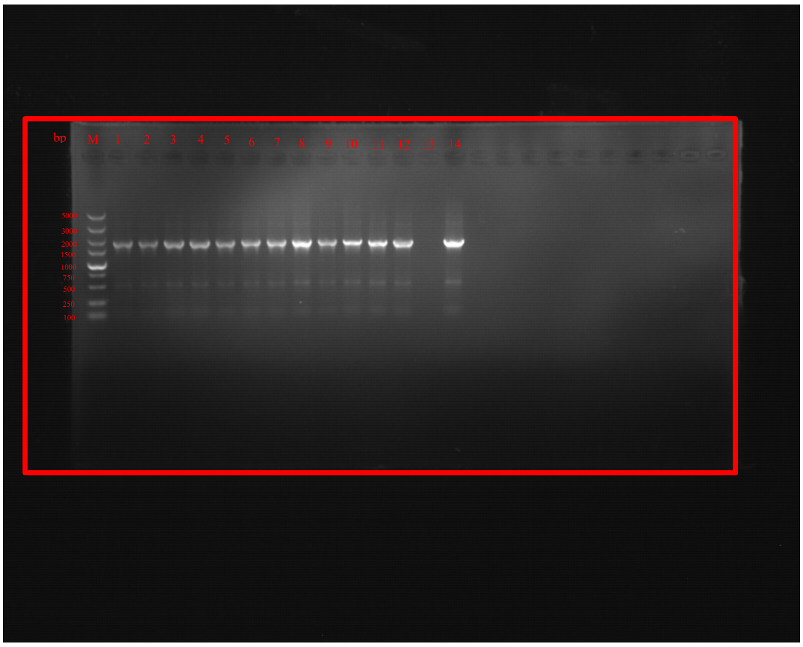

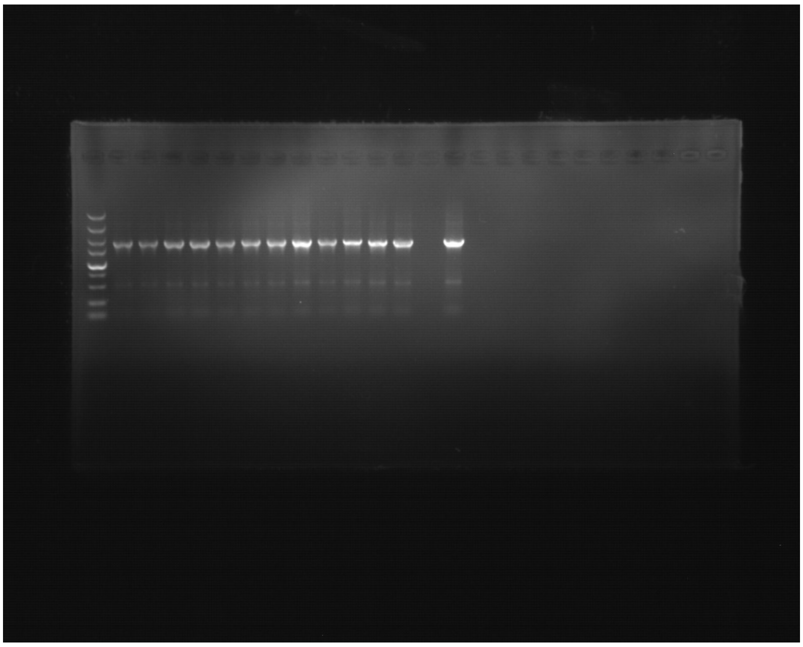

The original nucleic acid gel image corresponding to Supplementary Figure S2(A); Supplementary Figure S2 (A) depicts a cropped section of this image, encompassing wells 1 to

15 (as indicated by the red box). The order of sample addition, marker band sizes, and all graphic annotations remain consistent with those in the manuscript, and have been highlighted in red text.

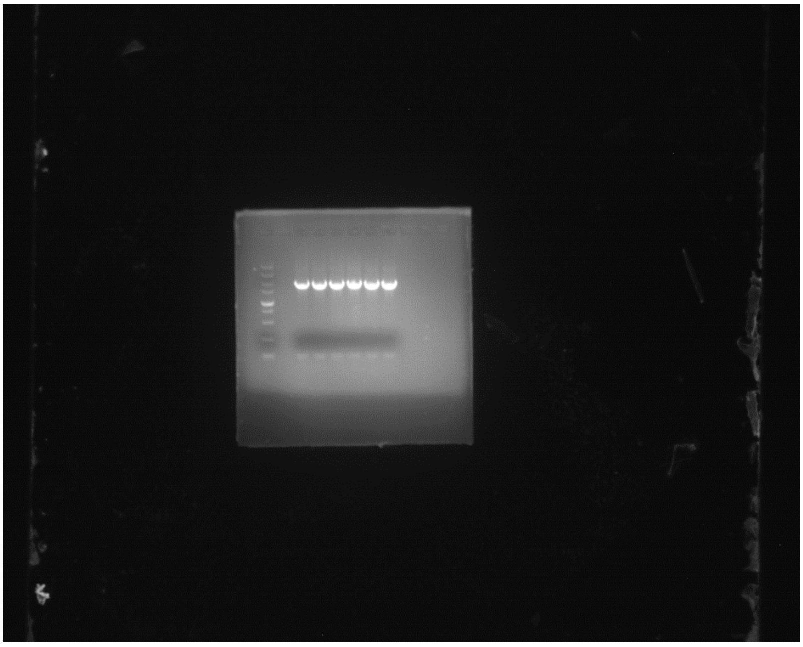

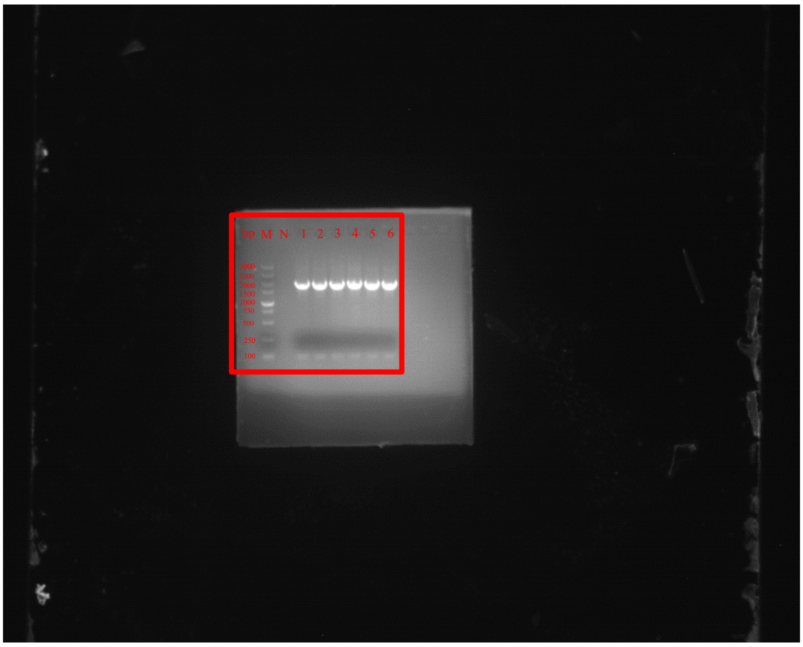

The original nucleic acid gel image corresponding to Supplementary Figure S2 (B); Supplementary Figure S2 (B) depicts a cropped section of this image, encompassing wells 1 to 8 (as indicated by the red box). The order of sample addition, marker band sizes, and all graphic annotations remain consistent with those in the manuscript, and have been highlighted in red text.
